# MITE Annotation and Landscape in 207 Plant Genomes Reveal Their Evolutionary Dynamics and Functional Roles

**DOI:** 10.1111/1755-0998.70041

**Published:** 2025-09-09

**Authors:** Jie Gao, Long‐Long Yang, Yi‐Ran Wang, Yue‐Yan Zhao, Yu Shi, Shuai‐Jie Wei, Ning Chen, Yu‐Lan Zhang, Wu‐Jun Gao, Shu‐Fen Li

**Affiliations:** ^1^ College of Life Sciences Henan Normal University Xinxiang China

**Keywords:** evolutionary dynamics, functional role, insertion time, MITE, MITE‐derived miRNAs

## Abstract

Miniature inverted‐repeat transposable elements (MITEs) are short, non‐autonomous class II transposable elements prevalent in eukaryotic genomes, contributing to various genomic and genic functions in plants. However, research on MITEs mainly targets a few species, limiting a comprehensive understanding and systematic comparison of MITEs in plants. Here, we developed a highly sensitive MITE annotation pipeline with a low false positive rate and applied it to 207 high‐quality plant genomes. We found over a 20,000‐fold variation in MITE copy numbers among species. The *Mutator* superfamily accounted for 41.5% of MITEs, whereas the *Tc1/Mariner* and *PIF/Harbinger* superfamilies expanded rapidly in monocots, particularly in Poaceae. Insertion time analysis revealed a general pattern of a single amplification wave, with initial insertions occurring around 30 million years ago (Mya) and peaking at 0–9 Mya. In addition, some species exhibited evidence of another ancient, slower expansion phase. In three representative families, we identified many more species‐specific MITE loci than shared MITE loci, underscoring MITEs' significant role in genome diversity. Phylogenomic analyses indicate that MITEs accumulated gradually and specifically during speciation, primarily through recent insertions rather than the retention of ancient elements. MITEs preferentially insert near genes and are often associated with enhanced gene expression. Furthermore, we identified 985 MITE‐derived miRNAs from 392 families across 56 species, mainly from *Mutator*, *Tc1/Mariner*, and *PIF/Harbinger*, targeting a variety of gene functions. This study enhances our understanding of the evolution and functional roles of MITEs in plants and provides a basis for exploring their function in further research.

## 1 Introduction

A comprehensive understanding of the evolutionary mechanisms that shape the complex genomes of eukaryotes necessitates an in‐depth examination of repetitive genomic sequences, particularly transposable elements (TEs) (Shapiro and von Sternberg 2005; Morgante et al. 2007). This necessity is particularly evident in higher plants, where TEs can constitute up to 91% of nuclear DNA (Sun et al. 2020) and play a significant role in the remarkable variation in genome size observed among different taxa (Springer 2015; Wong et al. 2019). Nonetheless, current research on TEs in plant genomes predominantly focuses on retrotransposons, which are Class I elements and are more prevalent. In contrast, Class II elements, namely DNA transposons, have received comparatively less attention.

The most numerous Class II elements in characterised plant genomes are miniature inverted‐repeat transposable elements (MITEs). MITEs are non‐autonomous TEs that are characterised by short length (typically 50–800 bp) and exhibit extremely high copy numbers. They are truncated derivatives of autonomous DNA transposons and contain terminal inverted repeats (TIRs) flanked by two short direct repeats known as target site duplications (TSDs) (Jiang et al. 2004). Due to the lack of functional transposase genes, their mobilisation relies on the transposase from related autonomous elements, which binds to the TIRs of the MITEs and facilitates both their excision and subsequent integration into novel genomic locations. Currently, based on the characteristics of TIR and TSD sequences of MITEs in plant genomes, they are primarily classified into the following five superfamilies: *Tc1/Mariner*, *PIF/Harbinger*, *Mutator*, *CACTA*, and *hAT* (Jiang et al. 2004; Fattash et al. 2013).

Since their discovery, MITEs have been identified in some organisms (Bureau and Wessler 1992, 1994; Casacuberta et al. 1998; Hu et al. 2000; Park et al. 2003). A database, P‐MITE encompassing MITEs from 41 plant genomes, was developed in 2014, revealing significant variability in their copy numbers and proportions across different plant species (Chen et al. 2014). It has been reported that MITEs tend to insert into gene‐rich regions and have the potential to regulate gene expression (Oki et al. 2008; Lu et al. 2012; Shen et al. 2017; Macko‐Podgórni et al. 2021; Zhang et al. 2024). For example, in *Oryza sativa* , the transcription factor TCP4 regulates grain size and tillering. In *japonica/geng* accessions, the presence of a *Tourist*‐like MITE in its promoter increases the methylation level of the promoter region, thereby suppressing *TCP4* expression and resulting in more tillers and shorter seeds (Zhang et al. 2024). Beyond their direct role in gene regulation, MITEs also serve as a source of certain miRNAs. These MITE‐derived miRNAs are instrumental in regulating target genes, thereby establishing novel “MITE–miRNA–target gene” regulatory networks. These networks play a crucial role in plant development, maintaining genomic stability, and mediating responses to environmental stimuli, such as stress resistance and hybridisation barriers (Kuang et al. 2009; Zhang et al. 2016; Cho 2018; Guo, Kuang, Tao, et al. 2022; Guo, Kuang, Zhao, et al. 2022; Zhan and Meyers 2023). These findings underscore the significant role of MITEs in influencing genomic variation, regulating gene expression, and impacting phenotypic traits, as well as contributing to the derivation of miRNAs. However, current research concerning the impact of MITEs on genome structure and function in plant genomes is predominantly focused on a limited number of species, notably *O. sativa* and *Zea mays* . The characterisation and comparative analysis of MITEs across a wide array of plant genomes are essential for advancing our understanding of genome evolution and function.

As sequencing technologies continue to advance, a number of MITE annotation tools have been developed, such as MITE‐Hunter, detectMITE, MITE Tracker and TIR‐Learner (Han and Wessler 2010; Ye et al. 2016; Crescente et al. 2018; Ou et al. 2019; Su et al. 2019). However, the short length of MITEs and the absence of distinctive structural domains pose significant challenges for current annotation software, which struggles to effectively balance false positives with sensitivity. In addition, almost all individual software is unable to accurately delineate the boundaries of MITEs. Therefore, the development of a MITE annotation pipeline that exhibits high sensitivity and minimises false positives is of great importance.

In this study, we established a pipeline by conducting a comparative analysis of the strengths and weaknesses of existing MITE annotation methods, with the aim of systematically identifying, classifying, and annotating MITEs across 207 high‐quality plant genomes. Based on these data, the abundance, characterisation, evolutionary dynamics, and functional roles of MITEs were examined.

## 2 Data and Methods

### 2.1 Acquisition and Quality Assessment of Plant Genome Data

The genome information on species with available genomes used in this study was obtained from the PubPlant online resource (Schwacke et al. 2025), and the genome data were acquired from multiple databases (Table S1). Because the genome quality can influence the annotation and comparative accuracy of genomic elements (Li, Sun, et al. 2023; Li, Zhang, et al. 2023), the angiosperm genomes were filtered according to chromosome‐level assembly, BUSCO score > 90%, contig N50 > 1 Mb, scaffold N50 > 20 Mb, and LTR Assembly Index (LAI) > 10. It was deemed sufficient if two of the latter three criteria were met, with any missing BUSCO and LAI values calculated by ourselves. Ultimately, 186 angiosperm genomes satisfying these criteria were selected, comprising three basal angiosperms, 23 monocots, four magnolias, and 156 eudicots. Furthermore, we also selected some representative non‐angiosperm genomes, including one gymnosperm, two pteridophytes, four bryophytes, and 14 algae. Thus, a total of 207 high‐quality plant genomes covering 65 families and 127 genera were used in this study (Table S1). Transcriptome data of six species were sourced from the NCBI Sequence Read Archive, with three biological replicates for each species (Table S2). For microRNA analysis, microRNA precursor and mature sequences were retrieved from the PmiREN2.0 (https://pmiren.com/) (Guo, Kuang, Tao, et al. 2022; Guo, Kuang, Zhao, et al. 2022) and miRBase (v22) (http://mirbase.org/) (Kozomara et al. 2019) databases, with only one duplicated miRNA retained. Within the 207 species, only 56 species from the above two databases were included. Therefore, miRNA analysis was performed in these 56 species. The number of miRNAs in each species is shown in Table S3.

### 2.2 Evaluation of MITE Annotation Software

To evaluate the annotation reliability of published software for identifying MITEs, we initially constructed a curated, non‐redundant MITE library of *Arabidopsis thaliana* . MITE sequences of *A. thaliana* were extracted from the Repbase (Jurka et al. 2005) and P‐MITE (Chen et al. 2014) databases and subsequently annotated using MITE Tracker (Crescente et al. 2018) and EDTA‐TIR‐Learner. Sequences exhibiting inaccurate boundaries in the MITE Tracker results underwent manual correction. The remaining candidates were manually checked for TIR and TSD to determine whether this element would be retained. Following the removal of redundant MITEs, the custom non‐redundant MITE library was employed to evaluate the performance of the MITE identification software, including MITE Tracker, EDTA‐TIR‐Learner, MUSTv2 and MiteFinderII, as well as the combined results of MITE Tracker and EDTA‐TIR‐Learner. The aforementioned non‐redundant MITE library was used as a reference library to conduct a comprehensive genome‐wide MITE identification using RepeatMasker (v4.1.1). Subsequently, the output from each software was employed as a library to perform an extensive genome‐wide search, referring to the “lib‐test.pl” script from the EDTA toolkit (Ou et al. 2019). Six parameters were calculated, including sensitivity, specificity, accuracy, precision, false positives, and the F1 measure, which is the harmonic mean of precision and sensitivity. This methodology was also applied to the monocot *Musa acuminata* and the fern *Selaginella moellendorffii*.

### 2.3 Identification and Classification of MITEs in Plant Genomes

Based on the evaluation results, we selected the combination of MITE Tracker and EDTA‐TIR‐Learner for the annotation of MITEs in plant genomes. Initially, MITE Tracker and EDTA‐TIR‐Learner were utilised independently to annotate MITEs. For MITE Tracker, the parameters were configured as follows: “‐‐tsd_min_len 2 ‐‐tsd_max_len 10 ‐‐mite_min_len 50 ‐‐mite_max_len 800”. Subsequently, Vsearch (Rognes et al. 2016) was employed with the parameters “‐‐iddef 1 ‐‐id 0.8” to cluster each MITE. Families with fewer than three copies were filtered out, resulting in the construction of an initial set of MITEs for each family. Since MITEs typically do not transpose with their flanking sequence (Crescente et al. 2018), only those elements exhibiting entirely distinct flanking sequences (50 bp on each side) compared to other MITEs within the same family were considered distinct individuals of that family. Subsequently, the sequence and annotation files of MITEs in each genome were compiled. However, due to the limitations of MITE Tracker in precisely identifying MITE boundaries, we extended the sequences by 50 bp upstream and downstream of each MITE to manually refine those with ambiguous boundaries and to categorise them into superfamilies according to the corrected TSD and TIR. Utilising this criterion, we manually corrected 51,121 families and selected MITEs with relatively complete TSDs and TIRs from each family to serve as the seed sequences. Although manually corrected boundaries facilitate the determination of the superfamily for the majority of MITEs, certain MITE families remain challenging to classify. For these ambiguous cases, we employed the DeepTE (Yan et al. 2020), which uses the convolutional neural network deep learning approach to assist in classification. Given the stringent boundary definitions of the EDTA‐TIR‐Learner identifications and the inclusion of superfamily information in its output, we conducted a comparative analysis between the position data of MITE Tracker software followed by manual correction and that produced by the EDTA‐TIR‐Learner software. MITEs exhibiting positional discrepancies of less than 100 bp between the two software outputs were regarded as identical, facilitating the elimination of duplicates.

**FIGURE 1:**
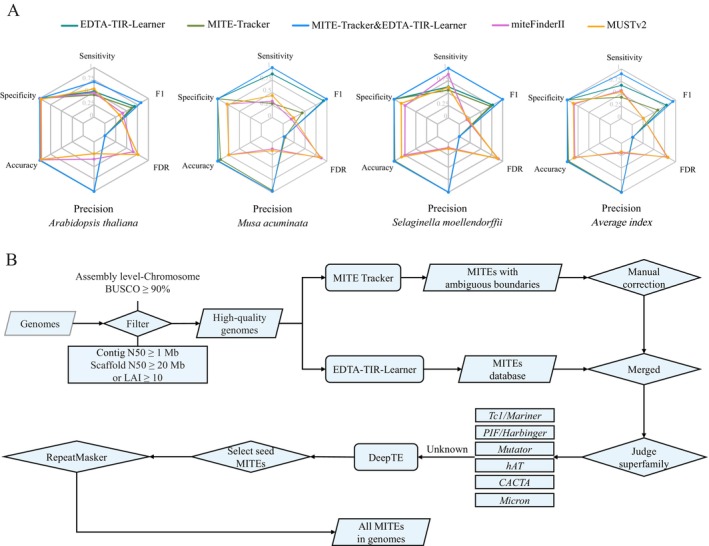
MITE annotation pipeline. (A) Evaluation of MITE annotation software. (B) Pipeline of MITE annotation.

To identify all MITE elements within the genome of each species, we employed RepeatMasker (v4.1.1) with parameters “‐s ‐x ‐no_is ‐nolow ‐cutoff 250 ‐div 15”, utilising the seed sequence library from each family as query sequences to perform a genome‐wide search. The detailed methodology is depicted in Figure 1.

Furthermore, we analysed the correlation between MITE characteristics (including cumulative MITE length, MITE copy number, and family number) and genome size, using the Spearman's correlation test. The significance (*p* value) and correlation coefficient (*r* value) were corrected using phylogenetic analysis. To infer the phylogenetic relationships among the 207 species, we constructed a phylogenetic tree based on genome‐wide distances using Mash (v2.3) (Ondov et al. 2016). Briefly, MinHash sketches of each genome were generated using the mash sketch command, and pairwise distances were calculated using mash dist. The resulting distance matrix was used to construct a phylogenetic tree via the Neighbour‐Joining algorithm. The resulting tree, saved in Newick format, was subsequently used for phylogenetic independent contrasts (PIC) analysis using the ‘pic()’ function from the R package ape.

Additionally, we also summarised the characteristics of the five major superfamilies and one special group of MITEs based on the EDTA‐TIR‐Learner software with minor modifications. The characteristics of their TSD and TIR are summarised in Table S4.

### 2.4 Estimation of the MITE Insertion Time

To investigate the amplification pattern of MITEs, multiple sequence alignments for each MITE family were generated utilising the MUSCLE program (v3.8.1551) (Edgar 2004). Subsequently, the consensus sequence for each MITE family was determined using a custom Python script, following a previously reported method (Xia 2018). The genetic distance (K value) of each MITE was estimated based on the consensus sequence, employing the ape package in R (Paradis et al. 2004) combined with the K80 model (Kimura's two‐parameter distance). The insertion time (*T*) of MITEs was estimated using the formula *T = K/2r* (Kimura 1980), where r represents the average substitution rate per synonymous site per year. Since different species across evolutionary branches exhibit varying r values, calculations were conducted according to the substitution rates provided previously (Table S5) (Li, Sun, et al. 2023; Li, Zhang, et al. 2023).

### 2.5 MITE Phylogenetic Analysis in Three Representative Families

The protein sequences of 24 species within the Rosaceae family, along with two outgroups, *Vitis vinifera* and *Morus notabilis*; 11 species from the Cruciferae family with one outgroup, *Carica papaya* ; and nine species from the Poaceae family with one outgroup *M. acuminata* , were analysed using OrthoFinder (v2.5.2) (Emms and Kelly 2015). The analysis aimed to identify sets of orthologous genes, resulting in the identification of 1369 single‐copy orthologous genes in the Rosaceae family, 1312 in the Brassicaceae family, and 90 in the Poaceae family. MAFFT (v7.487) (Katoh and Standley 2013) was employed to conduct multiple sequence alignments of single‐copy orthologous protein sequences. Subsequently, PAL2NAL (v14) was utilised to convert these protein alignments into codon alignments (Suyama et al. 2006). Further refinement of the alignments was achieved using Gblocks 0.91b (Castresana 2000). The phylogenetic tree was constructed from the trimmed alignment data using IQ‐TREE (v1.6.12) with parameters set to “‐bb 1000 ‐m TEST” (Nguyen et al. 2015) and visualised with Figtree (v1.4.4). Divergence times were estimated using MCMCTREE within the PAML v4.9j package (Yang 2007), calibrated with divergence times of the beginning and ending of evolutionary clades obtained from the TimeTree database (https://timetree.org/) (Hedges et al. 2006).

### 2.6 Detection of Insertion/Deletion MITE Polymorphic Sites

We selected five, six, and six species from the three families Rosaceae, Cruciferae, and Poaceae, respectively, to investigate the pattern of insertion/deletion MITE polymorphic sites. To enhance clarity, a flowchart was developed to visually represent this methodology (Figure S1). Specifically, we utilised five species from the genus within the Rosaceae family—namely, *Prunus armeniaca* , *Prunus sibirica*, *Prunus mandshurica*, *Prunus mume* , and *Prunus salicina* —to exemplify this process.

Initially, the full‐length MITEs were extended by 1000 bp both upstream and downstream. In cases where the insertion locus of a MITE was situated less than 1000 bp from the start of the chromosome, all available flanking sequences were utilised. The MITEs, along with their flanking sequences from *P. armeniaca* , were aligned with the genome sequences of the other four species using the BLASTN program. The alignment was performed under the following parameters: an E‐value threshold of less than 1e‐9 and identity greater than 80%. MITE hits that were in close proximity (within 1000 bp, the minimum length of a flanking sequence) were regarded as the same MITE insertion site. Based on these criteria, when the genomic region in the other four species is entirely aligned by flanking MITEs, either continuously or discontinuously, this MITE insertion site is defined as a shared MITE locus, and these MITE copies are considered as shared MITE copies. Subsequently, the genomes of the five species were subjected to BLASTN, with sequences containing shared MITE copies removed; fragmented results were still taken into account. In instances where the remaining four species could not be fully aligned using the MITEs containing flanking regions, and where the flanking sequences were identified in the remaining four species with an interspacing of less than 1000 bp, these insertion sites were classified as species‐specific MITE loci, and these MITE copies were considered as species‐specific MITE copies. Shared and species‐specific MITE copies were filtered using custom Python scripts for subsequent analysis.

### 2.7 Analysis of Insertion Loci and Adjacent Gene Features of MITEs in Genomes of Three Representative Families

To investigate the insertion characteristics of MITEs associated with genes in plant genomes, we analysed the genomes of species of the three representative families. The positions of the MITEs were compared with gene locations as indicated in the GFF annotation files. The number of MITEs inserted into exons, introns, and flanking regions of both 5000 bp upstream and 5000 bp downstream of genes was counted. The upstream and downstream regions were further subdivided into 500 bp intervals. Subsequently, we enumerated and visualised the number of MITEs inserted within the genes and each flanking region.

### 2.8 Analysis of the Effects of MITE Insertion on Gene Expression

The transcriptome data of six species of the three representative families underwent quality control using Fastp (v0.23.2) (Chen et al. 2018) to eliminate low‐quality reads. Subsequently, the processed reads were aligned to the reference genome using HISAT2 (v2.2.1) (Kim et al. 2019), and gene quantification was performed with FeatureCounts (v2.0.1) (Liao et al. 2014).

### 2.9 GO Enrichment Analysis

GO functional annotation of the protein sequences from the three representative families was carried out using the eggNOG‐mapper online platform (http://eggnog‐mapper.embl.de/) (Cantalapiedra et al. 2021). The OrgDb of each non‐model plant was constructed using the R program, based on the annotation results. GO enrichment analysis was performed using the clusterProfiler package (Yu et al. 2012).

### 2.10 Identification of MITE‐miRNA and MITE‐Derived miRNA

The miRNAs retrieved from the PmiREN2.0 and miRBase databases of 56 species were aligned with the MITEs of the same species by using BLASTN with parameters of ‐evalue 1e‐5 ‐perc_identity 80 ‐qcov_hsp_perc 50. MiRNA precursor sequences that exhibited alignment with MITEs for at least half of their length and demonstrated a sequence identity exceeding 80% were defined as MITE‐derived miRNAs. Accordingly, MITEs that could be aligned with MITE‐derived miRNAs were designated as MITE‐miRNAs.

### 2.11 Prediction of Target Genes of MITE‐Derived miRNA

The prediction of target genes for MITE‐derived miRNAs was conducted using the V2 scoring model of psRNATarget (https://www.zhaolab.org/psRNATarget/), with an expected value threshold set at 3 to ensure the retention of high‐confidence miRNA targets (Dai et al. 2018).

## 3 Results

### 3.1 Development of an Annotation Pipeline for MITEs in Plant Genomes

To accurately identify MITEs in plant genomes, we tested a variety of software across three species: *A. thaliana* , *M. acuminata* , and *S. moellendorffii*. The tools included MITE Tracker, EDTA‐TIR‐Learner, MUSTv2 and MiteFinderII, as well as a combined approach using EDTA‐TIR‐Learner and MITE Tracker followed by manual correction. The integration of EDTA‐TIR‐Learner and MITE Tracker demonstrated superior performance, achieving an average sensitivity of 90.31%, an average specificity of 99.92%, an average accuracy of 99.76%, an average precision of 99.06%, an average false positive rate of 0.93%, and an average F1 score of 93.87% (Figure 1A). This annotation pipeline significantly enhances the sensitivity of MITE annotation, effectively addressing the limitations of existing tools and offering a valuable methodology for annotating MITEs in plant genomes.

Utilising this method (Figure 1B), we identified a total of 667,333 full‐length MITEs, 54,998 MITE families, and 8,271,055 copies across 207 plant genomes. We manually corrected seed sequences of 51,121 families for further analysis. MITEs were detected in all studied genomes, with copy numbers ranging from 13 in the marine microalga *Bathycoccus prasinos* to 368,869 in the gymnosperm *G. montanum* .

### 3.2 Comparative Analysis of MITEs in Plant Genomes

**FIGURE 2:**
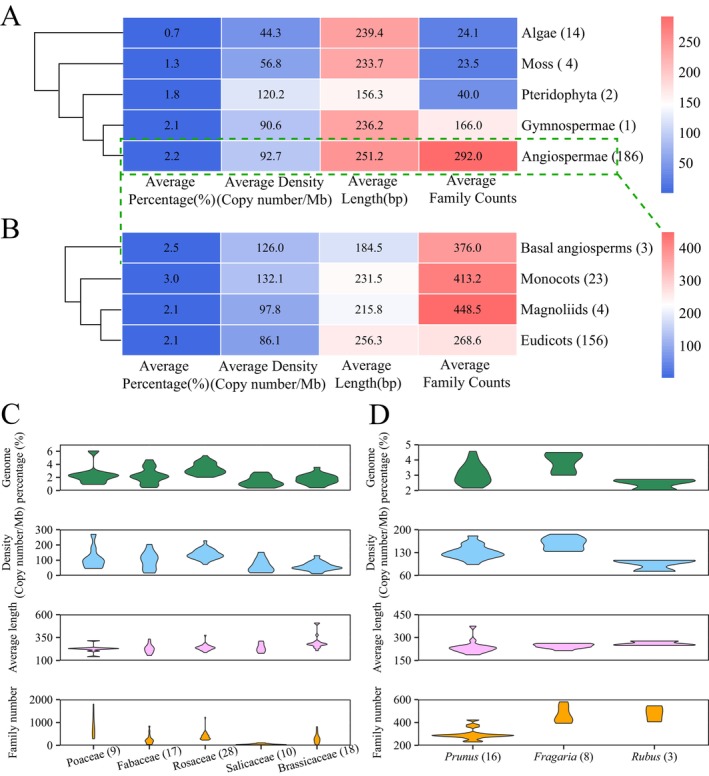
The content of MITEs in the genomes from different groups, families, and genera of plant species. (A) Different groups of the plant kingdom. (B) Various branches of angiosperms. (C) Diverse families. (D) Different genera within the same family. Different colours represent different indexes.

A comparative analysis of MITEs across angiospermae, gymnospermae, pteridophyta, moss, and algae was conducted, focusing on four key parameters: average proportion, average density, average length, and average family number. The average genome proportion of MITEs spanned from 0.7% to 2.2%, the average density per Mb ranged from 44.3 to 120.2, the average length varied between 156.3 and 251.2 bp, and the average family number ranged from 23.5 to 292 (Figure 2A). Significant variations were found in MITEs among the different plant groups. Notably, the average percentage, average length, and average family number of MITEs were found to be the highest in the 186 angiosperm species, whereas the average percentage and average density of MITEs were the lowest in algae. In conjunction with the principles of plant evolution, it is speculated that MITEs may have undergone varying degrees of expansion throughout the course of plant evolution. Subsequent research focused on the angiosperm groups exhibiting the highest MITE content, specifically basal angiosperms, monocots, magnoliids, and eudicots. Among these, MITEs in monocots and basal angiosperms appear to constitute the largest proportion of the genome, accounting for up to 3% and 2.5%, respectively (Figure 2B). However, a more in‐depth analysis revealed that *O. sativa* is an outlier within the monocots, exhibiting a MITE content as high as 6.04%, which significantly raises the average MITE content for the monocot group. Excluding *O. sativa* , the average MITE content among the remaining monocot species is reduced to 2.1%. Similarly, in basal angiosperms, *Amborella trichopoda* shows a relatively high MITE content of nearly 5%, which may give a misleading impression of high MITE abundance in this group. Overall, after excluding these exceptional species, the average MITE content and density among the four major angiosperm clades are comparable. For the average family counts, eudicots exhibit a markedly lower value compared to the other three groups (Figure 2B).

Next, we selected five families with a large number of species to analyse the four aforementioned indicators. The findings demonstrate that the average MITE proportion and density in Rosaceae are much higher than those in Fabaceae, Salicaceae, and Brassicaceae. Similar to the previous analysis, the high average MITE content and density observed in Poaceae are mainly due to the exceptionally high values in *O. sativa* . After excluding *O. sativa* , the remaining Poaceae species show significantly lower average MITE content and density than Rosaceae (Figure 2C). Furthermore, we conducted an analysis of MITE variations among three genera within the Rosaceae family: *Prunus*, *Fragaria*, and *Rubus*. The results clearly reveal that *Fragaria* possesses the highest MITE content, followed by *Prunus*, with *Rubus* exhibiting the lowest content (Figure 2D).

**FIGURE 3:**
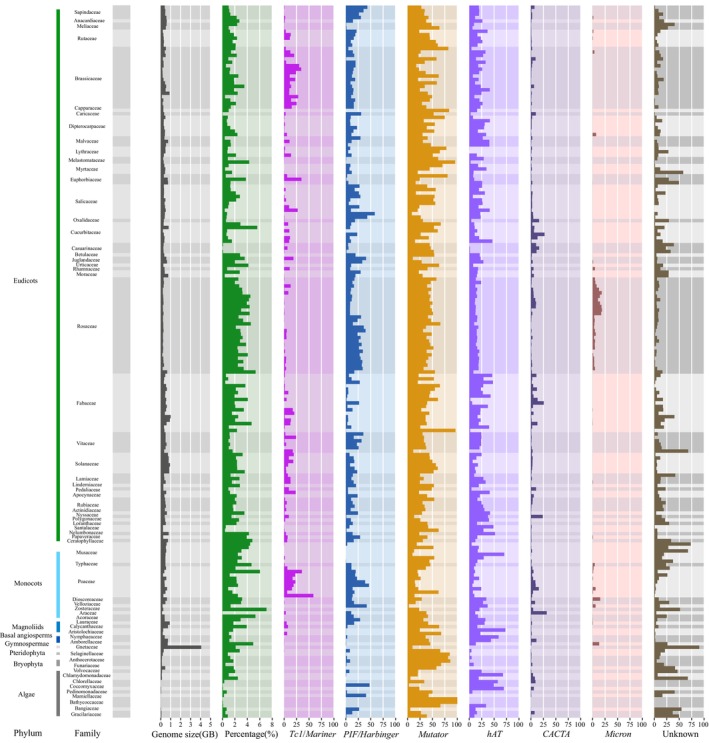
The distribution of different superfamilies of MITEs in the plant genomes.

The content of MITEs varies significantly across different plant groups, families, genera, and even species, with substantial differences observed even between closely related species. For example, MITEs account for 0.42% of the genome in *A. thaliana* , whereas in its close relative, *Arabidopsis lyrata* , they comprise 2.17% of the genome. These findings suggest that the evolutionary dynamics of MITEs appear to be independent of phylogeny and exhibit considerable variation between species (Figure 3).

We conducted a correlation analysis to examine the relationship between the total length, total copy number, and total family number of MITEs and genome size across various species. The analysis revealed predominantly significant positive associations between these examined variables and genome size. Notably, the correlation between genome size and cumulative MITE length was significant after phylogenetic correction (*r* = 0.60, *p* < 0.01). This correlation was weaker in angiosperms (*r* = 0.58, *p* < 0.01) yet more pronounced in non‐angiosperm species (*r* = 0.84, *p* < 0.01). A similar trend was observed for genome size and total MITE copy number, whereas the correlation with MITE family number was relatively weak (Figure S2).

To ascertain the MITE superfamily classification, we manually curated 51,121 MITE families, selecting sequences with perfect TSD and TIR as representative seeds. Through this approach, we identified the superfamilies of MITE families across various studied species and observed the evolutionary dynamic diversity of MITEs among different species (Figure 3). The results showed that the *Mutator* and *hAT* superfamilies are prevalent across nearly all species, whereas the *CACTA* superfamily is the least represented. The *Tc1/Mariner* and *PIF/Harbinger* superfamilies exhibit lower copy numbers in lower plants but have undergone significant expansion since the emergence of monocots. The *Micron* superfamily, initially identified in *O. sativa* , predominantly inserts into genomic microsatellite sequences (Akagi et al. 2001). In our analysis of the 207 species, the *Micron* superfamily is extensively present in the Poaceae and Rosaceae species, yet they are absent in other species, with the exception of a few individual species.

### 3.3 Analysis of the Insertion Times of MITEs

**FIGURE 4:**
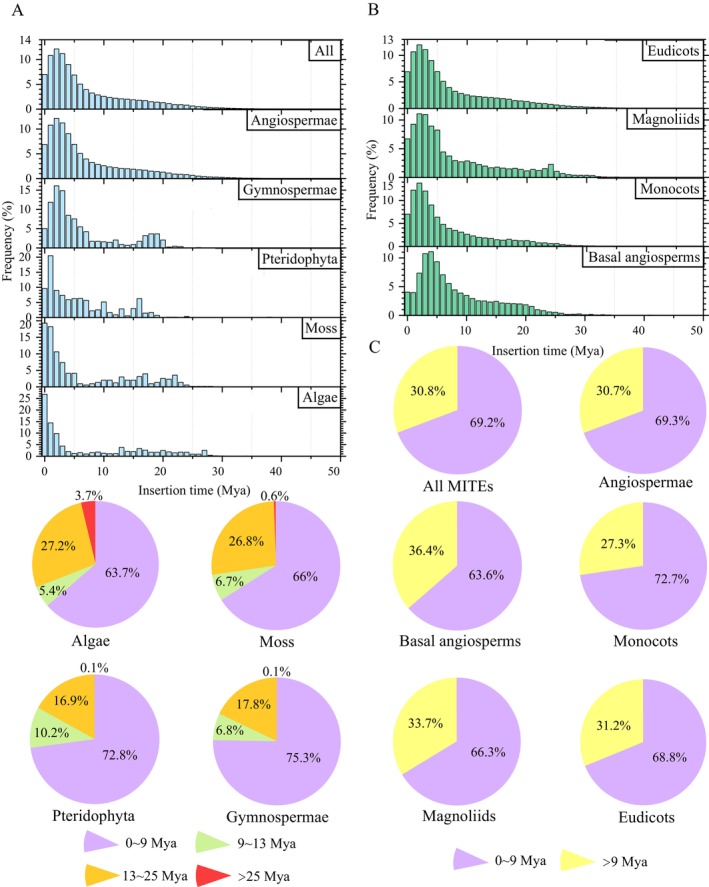
Insertion time and amplification pattern of MITEs in plant genomes. (A) Frequency distribution of amplification time of MITEs among different plant groups. (B) Frequency distribution of amplification time of MITEs in different branches of angiosperms. (C) The major amplification events of MITEs among different plant groups are shown in pie charts.

For the 207 species, the insertion times of MITEs across 54,998 families were estimated using the K80 model, and the results indicated that MITEs generally experienced a single expansion phase from 9 million years ago (Mya) to the present, during which 69.2% of MITEs were integrated into the genome (Figure 4A). Notably, a secondary small‐scale expansion occurred in gymnosperms, ferns, mosses, and algae between approximately 25 and 13 Mya, resulting in the insertion of an average of 22.2% of MITEs into the genome. In addition, a minority of MITEs, accounting for less than 1%, were inserted into the genome prior to 25 Mya. These findings indicate that MITEs remain largely inactive throughout most of their evolutionary history, experiencing rapid expansion only at specific intervals. In contrast to other plant groups, the expansion pattern of angiosperms appears relatively simple, characterised by a rapid expansion approximately 9 Mya, followed by a gradual integration into the host genome (Figure 4B,C).

### 3.4 Evolutionary Analysis of MITEs in the Genomes of Three Representative Families

To elucidate the relationship between the expansion of MITEs and species evolution, we selected three families of significant economic and agricultural importance: two dicotyledonous plant families, Rosaceae and Brassicaceae, and one monocotyledonous plant family, Poaceae. We conducted an analysis of the fundamental characteristics and evolutionary dynamics of their MITEs. A total of 221,609 full‐length MITEs were identified across the three families, encompassing 19,620 distinct families. A parallel trend was observed in both the quantity of MITEs and the diversity of families, with *O. sativa* exhibiting the highest content (Figure S3). Among the three families examined, species within the Rosaceae family demonstrated the least interspecies variation, with MITEs ranging from 2900 to 7886 and MITE families ranging from 260 to 580, alongside highly similar superfamily distributions. In contrast, species within the Brassicaceae and Poaceae families exhibited more pronounced interspecies variation, with MITE counts ranging from 228 in *Megadenia pygmaea* to 20,136 full‐length MITEs in *O. sativa* .

A genome‐wide search using RepeatMasker using the seed sequences of each family revealed that the proportion of the genome occupied by MITEs varied from 0.42% in *A. thaliana* to 6.04% in *O. sativa* (Figure S4A). The distribution of MITE superfamilies also exhibited distinct variations across the three families. In the Rosaceae family, *Mutator*‐like MITEs predominated, comprising an average of 45%, alongside a notable presence of *Micron*, averaging 7.7%. In contrast, the Brassicaceae and Poaceae families demonstrated a marked increase in the prevalence of *Tc1/Mariner* and *PIF/Harbinger* MITEs (Figure S4B). Subsequently, we conducted an in‐depth analysis of MITE expansion and their respective superfamilies within individual species to ascertain whether any particular superfamily exhibited unique expansion patterns in specific species. The results indicate that the MITEs across all species underwent a round of rapid expansion from about 9 Mya to the present. In addition, certain species exhibited an additional round of gradual expansion, notably observed in Rosaceae and Brassicaceae species (Figure S5). A comprehensive analysis of insertion time at the superfamily level revealed that different MITE superfamilies within the same species generally exhibited similar expansion patterns, although some obvious differences were also present. However, evolutionary trajectories of the same superfamily vary across different species, with certain superfamilies demonstrating transient, recent expansions in one species, while exhibiting sustained moderate activity in another (Figure S6). We focused on Rosaceae and Brassicaceae species which experienced two distinct rounds of MITE expansion. Notably, nearly all superfamilies participated in both rounds of expansion, indicating that the dual expansion events in Rosaceae and Brassicaceae species were collectively influenced by all MITE superfamilies, rather than being driven by a single specific superfamily.

### 3.5 Insertion/Deletion Polymorphism of MITEs in Plant Genomes of Different Families

**FIGURE 5:**
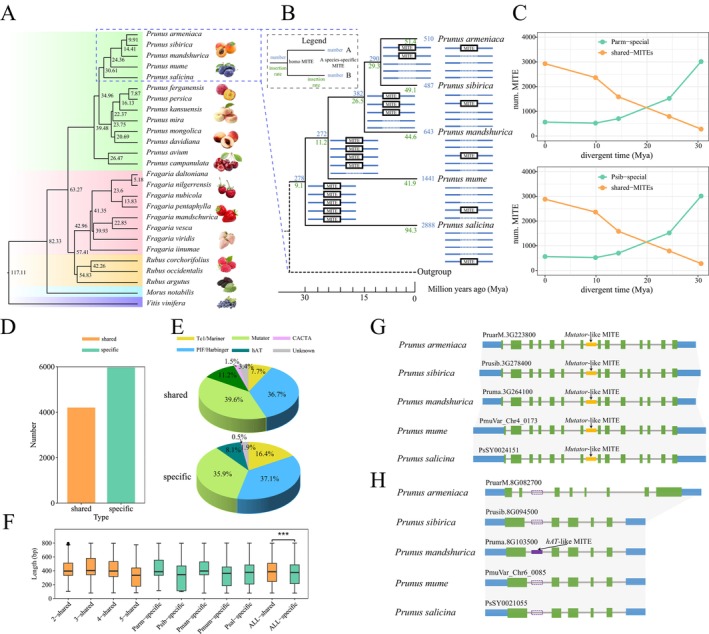
Evolution of MITEs during the differentiation of Rosaceae species. (A) Phylogenetic tree of Rosaceae species. (B) Phylogeny‐based statistics of orthologous MITEs. (C) Change patterns of shared and species‐specific MITE loci over divergence time, using *P. armeniaca* and *P. sibirica* as the reference species, respectively. (D) Statistics on the number of shared and species‐specific MITE copies. (E) The superfamily distribution of shared and species‐specific MITE copies. (F) Comparison between the length of shared and specific MITE copies. (G) Illustration of a shared MITE locus inserted within the corresponding introns of orthologous genes. (H) Illustration of a species‐specific MITE locus inserted into an intron of orthologous genes.

The insertion and deletion of MITEs inevitably lead to the presence/absence polymorphism in the host genome, specifically manifesting as insertion/deletion polymorphisms (Lu et al. 2012). To further elucidate the evolutionary relationships of MITEs on a phylogenetic scale, we identified orthologous MITEs in species belonging to the Rosaceae, Brassicaceae, and Poaceae families and constructed their polymorphism matrices. The divergence of Rosaceae species from the common ancestors of *V. vinifera* and *M. notabilis* occurred approximately 117.11 and 82.33 Mya, respectively (Figure 5A). We selected five *Prunus* species, representing relatively early evolutionary lineages, to investigate the insertion and deletion patterns of MITE polymorphic sites and to elucidate the dynamic evolutionary history of orthologous MITEs. In total, 7260 orthologous MITE loci were annotated, of which only 1291 (17.8%) were shared across species and represented 4202 copies, whereas the remaining 5969 loci (82.2%) were species‐specific, corresponding to 5969 copies (Figure 5C). The evolutionary dynamics of each MITE locus were mapped onto the phylogenetic tree. The results showed that the insertion frequency of shared MITE loci progressively increased from the ancestral nodes (9.1, 30.61 Mya) to the present nodes (29.3, 9.91 Mya) (Figure 5B). The proportion of species‐specific insertion increased with divergence time, whereas the proportion of shared MITEs showed an opposite trend (Figure 5C). The insertion frequency of species‐specific MITE sites in *P. salicina* (94.3) was significantly higher than that observed in the other four species (41.9–51.4). Notably, *Mutator*, *PIF/Harbinger*, and *Tc1/Mariner* represent the primary sources of these MITE copies (Figure 5D,E). In addition, the length of shared MITE copies is significantly greater than that of species‐specific MITE copies (*t*‐test, *p* < 0.001), indicating that longer MITEs are more likely to be retained, whereas newly inserted MITEs tend to be shorter (Figure 5F). MITE polymorphisms are observed in both genic and intergenic regions. We presented two examples of MITE polymorphisms located within intronic regions of genes to visualise the polymorphisms (Figure 5G,H). Furthermore, we performed sequence alignment of the shared MITE loci, which revealed a relatively high level of sequence similarity among these MITE sequences. The shared MITEs are most likely derived from ancestral insertions that have been vertically transmitted through evolutionary lineages. Thus, over long evolutionary timescales, these sequences have accumulated some mutations, and in some species, they have become fragmented MITE remnants (Figure S7).

We extended this analysis to the Brassicaceae and Poaceae families. MITE polymorphism analysis was conducted on six species within each family. Similar to the trend observed in Rosaceae, the number of shared MITE loci was significantly lower than that of the species‐specific MITE loci. The insertion frequency of the shared MITE loci exhibited a trend of being lower in ancestral nodes and higher in current nodes. MITEs exhibited species‐specific characteristics, and their accumulation gradually increased during the differentiation of species (Figures S8 and S9). Sequence alignment of the shared MITE loci also suggests that MITE sequences exhibit mutations and fragmentation (Figures S10 and S11). These findings indicate that the insertion and deletion of MITEs is a dynamic process: MITEs present at ancestral nodes can be mutated, degenerated, or even lost during the evolution process, whereas newly inserted MITEs represent the major source of species‐specific MITE copies.

### 3.6 Analysis of MITEs and Related Genes in the Genomes of Rosaceae, Brassicaceae and Poaceae

**FIGURE 6:**
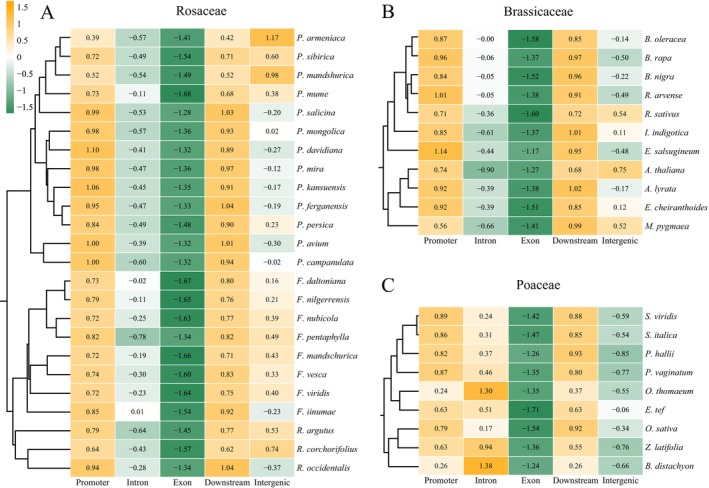
Normalised unit number density distribution of MITEs inserted into different genomic regions. (A) Rosaceae. (B) Brassicaceae. (C) Poaceae.

An analysis of the distribution of MITEs in different genomic regions revealed that, with few exceptions, MITEs preferentially inserted into promoter and downstream regions, followed by intergenic regions and introns, with exon regions being the least frequently targeted (Figure 6). This pattern is consistent with findings from previous studies (Liu et al. 2019). Therefore, MITEs exhibit a preferential insertion into gene‐flanking regions, particularly within the promoter region, which implies that MITEs preferentially insert into open chromatin and can contribute transcription factor binding sites or enhancer elements, thereby acting as potentially *cis*‐regulatory modules for adjacent genes. It is noteworthy that nearly all species exhibited a peak in the MITE quantity within the 500–1000 bp region flanking the gene. This peak gradually diminished on both sides, with no significant differences observed between the upstream and downstream regions (Figure S12). We speculate that this peak may represent a MITE insertional hotspot, which may be attributed to the enrichment of gene regulatory elements within the 500–1000 bp region, where MITE insertions are more likely to influence gene expression.

**FIGURE 7:**
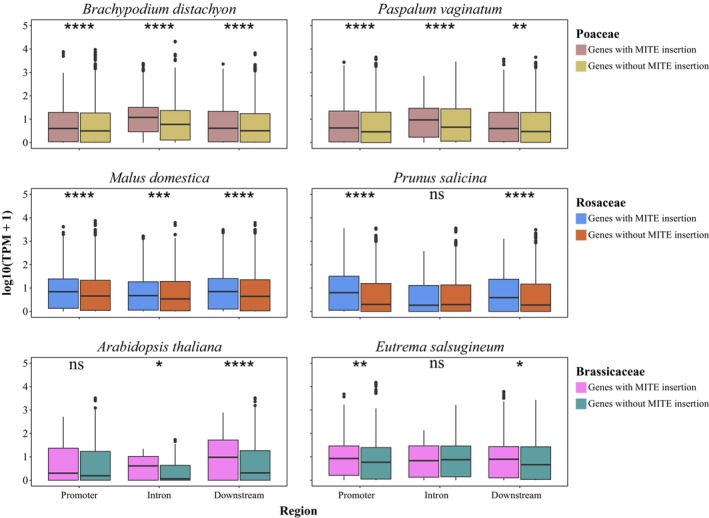
Effect of MITE insertions on gene expression in representative species of Rosaceae, Brassicaceae, and Poaceae (p**** < 0.0001; p*** < 0.001; p** < 0.01; p* < 0.05; ns indicates no significant difference).

To investigate the relationship of MITE insertions and gene expression, we performed gene expression analyses across six species from the three families. The results demonstrated that genes with MITE insertions in the promoter, intron, and downstream regions exhibited significantly elevated expression levels compared to those without such insertions (Figure 7). This effect was most pronounced in *Brachypodium distachyon* , *Paspalum vaginatum* , and *Malus domestica* (*p* < 0.001), providing further evidence that MITE insertions preferentially occur in open chromatin regions where the genes are highly expressed. It is also possible that some of the MITE insertions can regulate the expression of adjacent genes, predominantly by enhancing gene expression.

GO enrichment analysis was conducted on genes containing MITE insertions within promoter, intronic, and downstream regions to elucidate the functional tendencies associated with MITE‐related genes. In general, the functions of genes harbouring MITE insertions are diverse, including roles in transport, synthesis, metabolism, and stress response (Figure S13). Notably, genes with MITE insertions in intronic regions demonstrated evident patterns of functional regularity. Within the top 30 terms exhibiting the highest frequency, Poaceae species were predominantly enriched in terms pertaining to genomic structure, and Rosaceae species were frequently associated with terms related to transport, metabolism, and structure, whereas Brassicaceae species did not show widespread enrichment (Figure S13B). In contrast, the functional enrichment analysis of genes with MITE insertions in the promoter and downstream regions demonstrated species‐specific functional diversity, with a primary focus on terms such as ‘transport’, ‘synthesis’, and ‘metabolism’ (Figure S13A,C).

### 3.7 Comparative Analysis of MITE‐Derived miRNAs in 56 Plant Species

**FIGURE 8:**
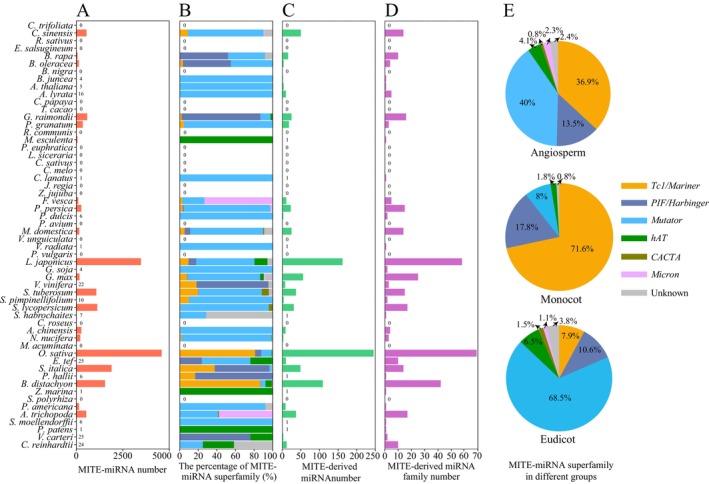
Characteristics of MITE‐miRNA and MITE‐derived miRNAs in 56 species. (A) The number of MITE‐miRNA. (B) The proportion of each MITE‐miRNA superfamily. (C) The number of MITE‐derived miRNAs. (D) The number of MITE‐derived miRNA families. (E) The superfamily distribution of MITE‐miRNA in different plant groups.

MITEs are capable of generating a substantial number of miRNAs. Analysis of the characteristics of MITE‐derived miRNAs and their associated MITEs in 56 species revealed that *O. sativa* possesses the largest number of MITE‐derived miRNAs (Figure 8A). This abundance correlates with the highest copy number of MITE copies observed in *O. sativa* . Additionally, MITE‐derived miRNAs have not been found in some species, such as those in the Cucurbitaceae family. This phenomenon could be attributed to two main factors: (1) intrinsic genomic differences among plant species may result in differential production of miRNA enzyme loci, thereby leading to a reduced number of miRNAs in certain species (Chen et al. 2021); (2) the genomes may not have undergone extensive MITE amplification events, resulting in a limited number of MITE copies and, consequently, a diminished miRNA count. The majority of MITE‐miRNAs are associated with the *Mutator*, *Tc1/Mariner*, and *PIF/Harbinger* superfamilies (Figure 8B). The abundance and family distribution of MITE‐miRNAs exhibit similar patterns (Figure 8C,D). Among the 56 species analysed, 52 belong to angiosperms, with the *Tc1/Mariner*, *PIF/Harbinger*, and *Mutator* superfamilies representing the predominant components. This suggests that these three superfamilies are the primary sources of MITE‐miRNAs in angiosperm genomes (Figure 8E). In monocots, 71.6% of MITE‐derived miRNAs originate from the *Tc1/Mariner* superfamily, whereas in eudicots, 68.5% are derived from the *Mutator* superfamily, with *PIF/Harbinger* elements closely following these two. This pattern indicates a biased expansion of superfamilies between monocots and eudicots, leading to the derivation of miRNAs.

For the three major superfamilies of MITE‐derived miRNAs in angiosperms—*Tc1/Mariner*, *PIF/Harbinger*, and *Mutator*—we conducted a comparative analysis of the lengths of MITE‐miRNAs and non‐MITE‐miRNAs, using all MITEs as a control group. The results showed that the length distribution of non‐MITE‐miRNAs aligns with that of all MITEs, whereas MITE‐derived miRNAs exhibit a divergent pattern (Figure S14). Specifically, within the *PIF/Harbinger* and *Mutator* superfamilies, MITE‐miRNAs demonstrate two or three distinct peaks at around 150, 350, and 500 bp, with more concentrated length distributions. In contrast, non‐MITE‐miRNAs display more peaks, reflecting a more dispersed length distribution. The average length of MITE‐miRNAs is 272 bp, in contrast to the 331 bp observed in non‐MITE‐miRNAs. This suggests that the proliferation of shorter MITEs is more likely to produce miRNAs, potentially influencing miRNA biogenesis within the genome.

Additionally, we conducted a comparative analysis of the free energy between MITE‐miRNAs and non‐MITE‐miRNAs, focusing on species with a greater prevalence of MITE‐miRNAs for analysis. The normalised minimum free energy (NMFE) of MITEs reflects the stability of their hairpin structures, which has a significant impact on miRNA production (Huang et al. 2022). Comparative analysis revealed that the peak of NMFE for MITE‐miRNAs is significantly lower than that for non‐MITE‐miRNAs. This suggests that MITE‐miRNAs have a greater propensity to form hairpin structures and are more predisposed to produce miRNAs (Figure S15).

### 3.8 GO Functional Analysis of Soybean MITE‐Derived miRNA Target Genes

Across the 56 species, no consistent pattern was found in the GO functional enrichment of target genes associated with MITE‐derived miRNAs. However, in *Glycine max* , one of the most frequently enriched functions identified was related to growth and development (Figure S16A), with significant enrichment observed in GO terms such as “photoperiodism”, “photomorphogenesis”, “flowering” and “cell division” (Figure S16B). We identified a MITE‐derived miRNA, miR10424, along with its target gene, *GmW82.07G059700* (Figure S16C), which has been confirmed to encode an inhibitor of the PHYA 105 protein. This gene plays a significant role in *G. max* development by undergoing self‐truncation, thereby enhancing seed yield under conditions of high‐density planting (Li, Sun, et al. 2023; Li, Zhang, et al. 2023). To further illustrate the regulatory role of MITEs in *G. max* growth and development, we constructed a model depicting the “MITE‐miR10424‐PHYA 105” pathway (Figure S17). Following a large‐scale proliferation of MITEs in *G. max* , a subset of transcriptionally active MITEs might undergo selective conversion into miRNAs, thereby expanding the miRNA repertoire and probably enhancing the gene regulatory capacity of the host genome (Figure S17).

## 4 Discussion

### 4.1 The Robust MITE Annotation Pipeline Is Crucial for Comparative Analysis in Multiple Organisms

Plant genomes contain a large number of MITE copies, which significantly impact genome variation, gene regulation, and small RNA production (Feschotte and Mouches 2000; Pegler et al. 2023; Zhang et al. 2024). However, current research on MITEs has mostly been limited to a few species like *O. sativa* (Lu et al. 2012), *Triticum aestivum* (Keidar‐Friedman et al. 2018), *Daucus carota* (Macko‐Podgórni et al. 2021), and citrus species (Liu et al. 2019). The limited data restrict a thorough understanding and comparison of MITEs across a wide range of plants. In addition, the short internal sequences of MITEs, lacking open reading frames, hinder accurate detection. Most methods struggle with high false positive rates, fail to correctly differentiate superfamilies, and cannot manage large genomes effectively (Crescente et al. 2018). Consequently, we developed a MITE annotation pipeline with high sensitivity and low false positives and manually refined the boundaries of certain MITEs to accurately ascertain their superfamily classification. The quality of the genome significantly influences the annotation of transposons. To minimise the impact of technical inaccuracies on the annotation outcomes of MITEs, we screened plant genomes based on criteria such as contig N50, scaffold N50, LAI, BUSCO, and assembly at the chromosome level. Finally, the MITEs in 207 high‐quality plant genomes were systematically annotated and analysed using our method, which clarifies MITE boundaries better than MITE Tracker and addresses incomplete annotations of EDTA‐TIR Learner. Manual corrections enhance reliability, making it a reliable MITE annotation approach.

### 4.2 The Evolution of MITEs Accelerates Genome Structure Diversity in Plants

Different plant groups exhibit a variety of MITE diversity, with angiosperms exhibiting a notably higher content of MITEs compared to other groups. This indicates that MITEs may contribute to the rapid adaptation of angiosperms during their evolutionary process. In fact, there is a significant positive correlation between MITE content and genome size, suggesting that the amplification of MITEs is a driving contributing factor to genome expansion (Eriksson et al. 2022). However, certain notable exceptions exist. For example, MITEs constitute 6.04% of the genome in *O. sativa* , despite its relatively modest genome size of 377.6 Mb. In contrast, the genome of *C. papaya* , which is comparable in size at 351 Mb, contains only 0.06% MITEs. We speculate that this discrepancy may be attributed to the evolution process of MITEs. Specifically, the TIR region, which serves as transposase binding sites in their ancestral autonomous DNA transposons, may have undergone mutations or recombination events during the evolution of MITEs. These alterations potentially impede the encoding of functional transposases. Consequently, the “cross‐mobilisation” mechanism is reduced, resulting in a decreased derivation of MITEs (Jiang et al. 2003). Furthermore, it should be noted that large genomes were not included in this study due to challenges related to assembly quality and the difficulty of manually curating a large number of MITEs. Therefore, further investigations are required to determine whether MITEs may serve as a driving force in the evolution of large genomes. Through the manual correction of the representative sequence for each family and the determination of its superfamily, it was observed that *Mutator* and *hAT* MITEs are prevalent across nearly all species, whereas *CACTA* is the least common. Additionally, *Tc1/Mariner* and *PIF/Harbinger* are less prevalent in lower plants but have undergone significant expansion since the emergence of monocots. Notably, *Micron*, a type of MITE initially discovered in *O. sativa* , tends to insert into (TA)n repeat sequences, with a smaller proportion integrating into (CA)n or (GT)n repeat sequences. Given the inherent instability of the (TA)n microsatellite regions, the insertion of MITEs into these vulnerable regions may enhance their stability, thereby conferring potential advantages to the host genome (Lin et al. 2021). In this study, *Micron*‐like MITEs were observed to be extensively distributed across the Poaceae and Rosaceae species. The underlying cause of this phenomenon warrants further investigation. Previous studies have suggested that *Micron*‐like MITEs are likely derivatives of *Mutator*‐like DNA transposons. These elements may have adopted a damage‐limitation strategy during their co‐evolution with host genomes, favouring insertion into microsatellite regions. This strategy could facilitate evasion of genomic control mechanisms, thereby enabling extensive proliferation (Lin et al. 2021). MITEs primarily experienced a round of rapid and large‐scale expansion, which was completed approximately 20–30 Mya, with a peak initially occurring between 9 and 10 Mya. During this period, angiosperms exhibited significant diversity, and the number of species increased dramatically (Lu et al. 2018). This large‐scale insertion occurrence is referred to as a transposon “burst” event (Kidwell and Lisch 2001). These events, together with other genetic material changes, are believed to contribute to the rapid adaptation to environmental changes of angiosperms during their evolutionary processes (Li, Sun, et al. 2023; Li, Zhang, et al. 2023).

Employing a phylogenomics‐based approach, we identified both shared and species‐specific MITEs across three representative plant families to investigate their evolutionary dynamics in closely related species. The results demonstrated that species‐specific MITE loci are more abundant than shared loci. Moreover, many shared MITE copies exhibit signs of degeneration, including mutations, insertions, and sequence divergence. Notably, the relative insertion frequency of shared MITE loci declines from present nodes to ancestral nodes. These observations suggest that MITE insertion and deletion is a highly dynamic process, with ancestral insertions progressively lost in different lineages/species over evolutionary time. The retention of MITEs represents the interplay between MITE insertions and the filtering processes of natural selection and genetic drift. Patterns of MITE insertion/deletion polymorphisms indicate that species‐specific MITEs mainly represent recent insertions, whereas shared MITE copies reflect long‐term retention since species divergence. The higher proportion of species‐specific MITEs indicates a bias towards recent insertions and a relatively low frequency of long‐term retention events. The analysis of MITE insertion time further supports this trend. A significant 69.2% of MITE insertions happened from about 9 Mya to present, with a smaller 22.2% expansion in gymnosperms, ferns, mosses, and algae between 25 and 13 Mya. Less than 1% of MITEs were inserted before 25 Mya. Thus, most MITEs are recent insertions. These findings collectively suggest that MITE accumulation has primarily occurred through recent insertions rather than through the retention of ancient insertions. Additionally, the insertion rate of species‐specific MITEs exhibits variability across different species. In the Brassicaceae family, it was evident that the insertion of species‐specific MITEs exhibited a significant correlation with the overall MITE content within the species. This indicates that the amplification patterns of MITEs have a direct impact on the number of species‐specific loci. These findings suggest that MITE insertions have substantially contributed to genome polymorphism and play a crucial role in genome diversity and genome structure variation.

It should be emphasised that the estimation of MITE expansion periods in this study is based on sequence divergence from family consensus sequences. Although this approach is commonly used and works reasonably well for individual families, consensus sequences are abstract constructs that likely never existed in nature (Storer et al. 2022; Wang et al. 2025). Consequently, applying this method at a large scale may introduce potential artefacts or lead to misinterpretations. Therefore, the inferred timing of MITE insertions should be regarded as indicative of general evolutionary trends rather than as precise estimates and cannot be directly compared with the evolutionary timescales of the host species.

### 4.3 The Distribution Patterns of MITEs and Their Impact on Gene Regulation

Beyond their role in genome evolution, accumulating evidence suggest that MITEs can regulate gene expression (Oki et al. 2008; Shen et al. 2017). Analysis of the genomic locations of MITEs within the species of the three representative families demonstrated a preferential insertion of these elements into gene‐flanking regions, particularly within 500–1000 bp of the genes, suggesting that MITEs primarily target open chromatin, which is consistent with previous findings (Macko‐Podgórni et al. 2019). A comparative transcriptomic analysis was conducted to assess gene expression levels across four species, focusing on genes with MITE insertions in promoters, introns, and downstream regions, as well as genes lacking such insertions. The results showed that most of the genes associated with MITE insertions have relatively high expression levels. It is possible that MITEs insert preferentially in the vicinity region of genes with elevated expression, presumably as a consequence of an open chromatin configuration. However, it is also possible that some of the MITE insertions described here may actually modify gene expression, as some genes associated with MITE insertions have low expression, and some have high expression, compared with the paralogous genes away from MITEs. In fact, examples of MITE elements potentially altering gene expression have accumulated over the years (Naito et al. 2009; Zheng et al. 2019; Yin et al. 2020; Castanera et al. 2021; Niu et al. 2022). The mechanisms by which MITEs regulate gene expression are diverse. For example, the insertion of an *hAT*‐like MITE upstream of the *GLO1* start codon upregulates the promoter activity of *GLO1* by providing several cis‐regulatory elements, enhancing *GLO1* expression (Yang et al. 2024). In addition, in the citrus model plant *Fortunella hindsii* , a 786 bp *hAT*‐like MITE was identified in the *FhiS2‐RNase* promoter region. The insertion of this MITE inhibited the expression of *S‐RNase*, exhibiting a self‐compatibility (SC). Transgenic experiments indicated that the deletion of this 786 bp MITE restored the expression of *FhiS2‐RNase* and restored the self‐incompatibility trait (Hu et al. 2024). In addition, MITEs can also regulate gene expression by other mechanisms, such as posttranscriptionally producing new transcriptional variants and generating small RNAs (Kum et al. 2015; Xin et al. 2019; Campo et al. 2021). These findings collectively suggest that MITEs preferentially integrate into the vicinity of genes and have the potential for modulating downstream gene expression through various mechanisms and consequently altering plant phenotypes.

### 4.4 Potential Regulatory Roles of MITE‐Derived miRNAs and Their Target Genes in Modulation of Plant Phenotypes

MITEs may serve as a potential source of genomic miRNAs and contribute to the regulation of target genes. We identified several factors influencing MITE‐miRNA production, including MITE length, copy number, and normalised minimum free energy. Additionally, MITEs are capable of generating miRNAs that predominantly belong to three superfamilies: *Mutator*, *Tc1/Mariner*, and *PIF/Harbinger*. This observation suggests that the diversification of these MITE superfamilies may have facilitated miRNA production in angiosperms. Since the emergence of angiosperms, they have surpassed other plant groups in species richness and ecological dominance (Rubinstein et al. 2010; Magallon et al. 2013). The expansion of MITEs drives the diversity of intrinsic traits in angiosperms through multifaceted interactions with external forces (Jeong et al. 2023). In the case of *G. max* , we identified a number of MITEs that give rise to miRNAs, some of which potentially regulate genes involved in growth and development, indicating that MITE‐derived miRNAs have the potential to enhance the environmental adaptability of *G. max* . It is plausible that the integration of novel miRNAs, generated by MITEs, into gene regulatory networks in a lineage‐specific manner could lead to the diversification of homologous gene expression, thereby facilitating the emergence of new phenotypes (Wu et al. 2015).

## Author Contributions

Conceptualisation, S.‐F.L. and W.‐J.G.; methodology and formal analyses, J.G., L.‐L.Y., and Y.‐R.W.; investigation, all authors; writing – original draft, J.G.; writing – review and editing, all authors; project administration, S.‐F.L. and W.‐J.G.; funding acquisition, S.‐F.L. and W.‐J.G. All authors have read and approved the final manuscript.

## Conflicts of Interest

The authors declare no conflicts of interest.

## Supporting information


**Appendix S1:** men70041‐sup‐0001‐Supinfo.pdf.

## Data Availability

All data used in this study were downloaded from publicly available databases. Detailed information on the species and their corresponding accession numbers from all datasets is provided in Table S1. The seed sequences of the identified MITE families with manual curation, as well as the MITE‐derived miRNA sequences, were deposited in the Dryad digital repository with the DOI of 10.5061/dryad.bg79cnpns.
