# Supplementary material for: MITE Annotation and Landscape in 207 Plant Genomes Reveal Their Evolutionary Dynamics and Functional Roles
Source: Mol Ecol Resour. 2025 Sep 9;25(8):e70041. doi: 10.1111/1755-0998.70041 (PMC12550479; doi:10.1111/1755-0998.70041)
Supplement: Supplementary file 1 — Appendix S1: men70041‐sup‐0001‐Supinfo.pdf. [file MEN-25-e70041-s001.pdf]

MOLECULAR ECOLOGY RESOURCES

Supplemental Information for:

MITE Annotation and Landscape in 207 Plant Genomes Reveal Their Evolutionary Dynamics and Functional Roles

Jie Gao, Long-Long Yang, Yi-Ran Wang, Yue-Yan Zhao, Yu Shi, Shuai-Jie Wei, Ning Chen, Yu-Lan Zhang, Wu-Jun Gao*, Shu-Fen Li*

Table of Contents:

Figure S1	Page 1
Figure S2	Page 2
Figure S3	Page 3
Figure S4	Page 4
Figure S5	Page 5
Figure S6	Page 6
Figure S7	Page 7
Figure S8	Page 8
Figure S9	Page 9
Figure S10	Page 10
Figure S11	Page 11
Figure S12	Page 12
Figure S13	Page 13
Figure S14	Page 14
Figure S15	Page 15
Figure S16	Page 16
Figure S17	Page 17
Table S1	Page 18 - Page 26
Table S2	Page 27

MOLECULAR ECOLOGY RESOURCES

**Table S3**	Page 28 - Page 30
**Table S4**	Page 31
**Table S5**	Page 32

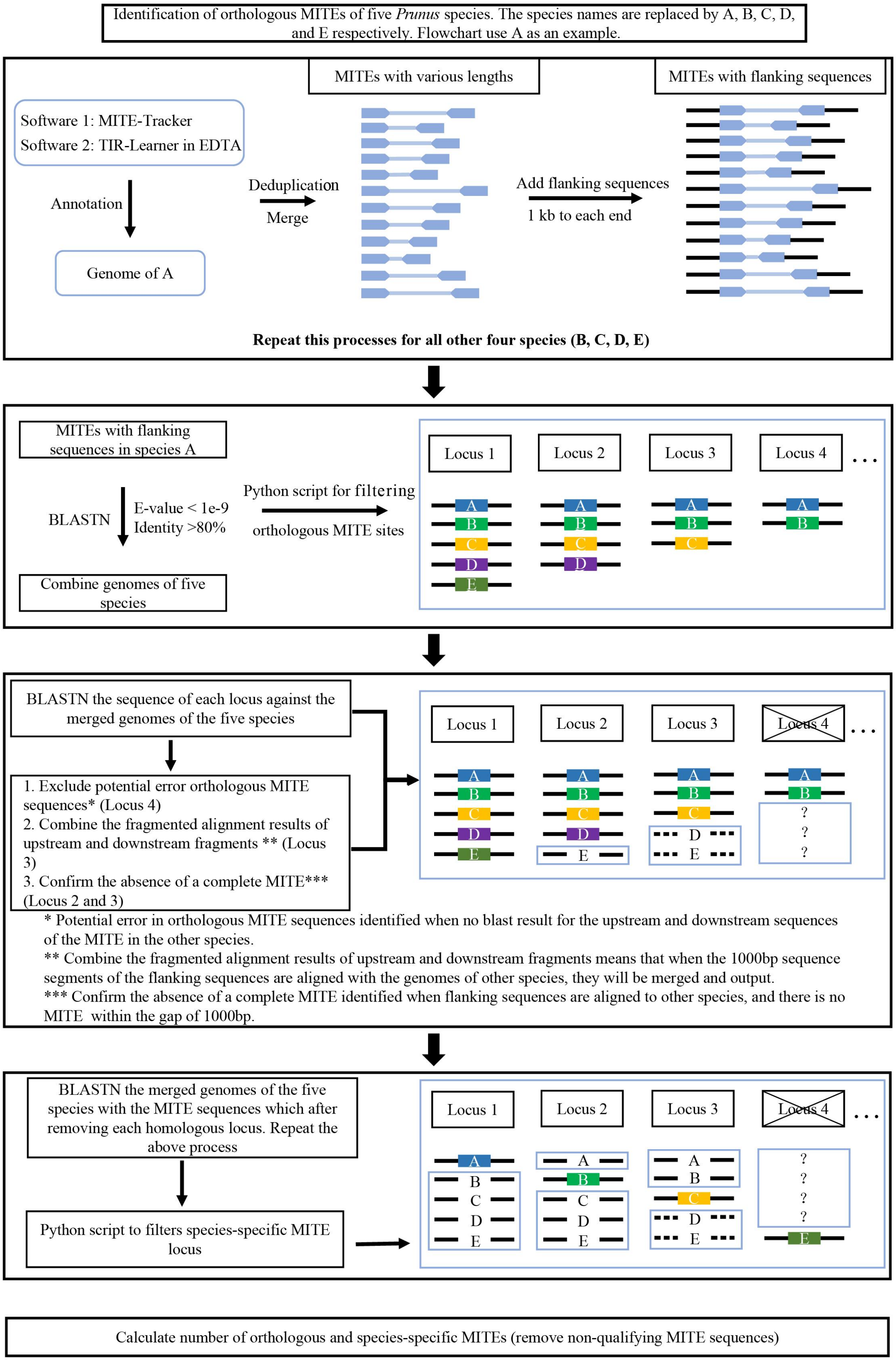

Figure S1 Flow chart for identification of orthologous MITE sites.

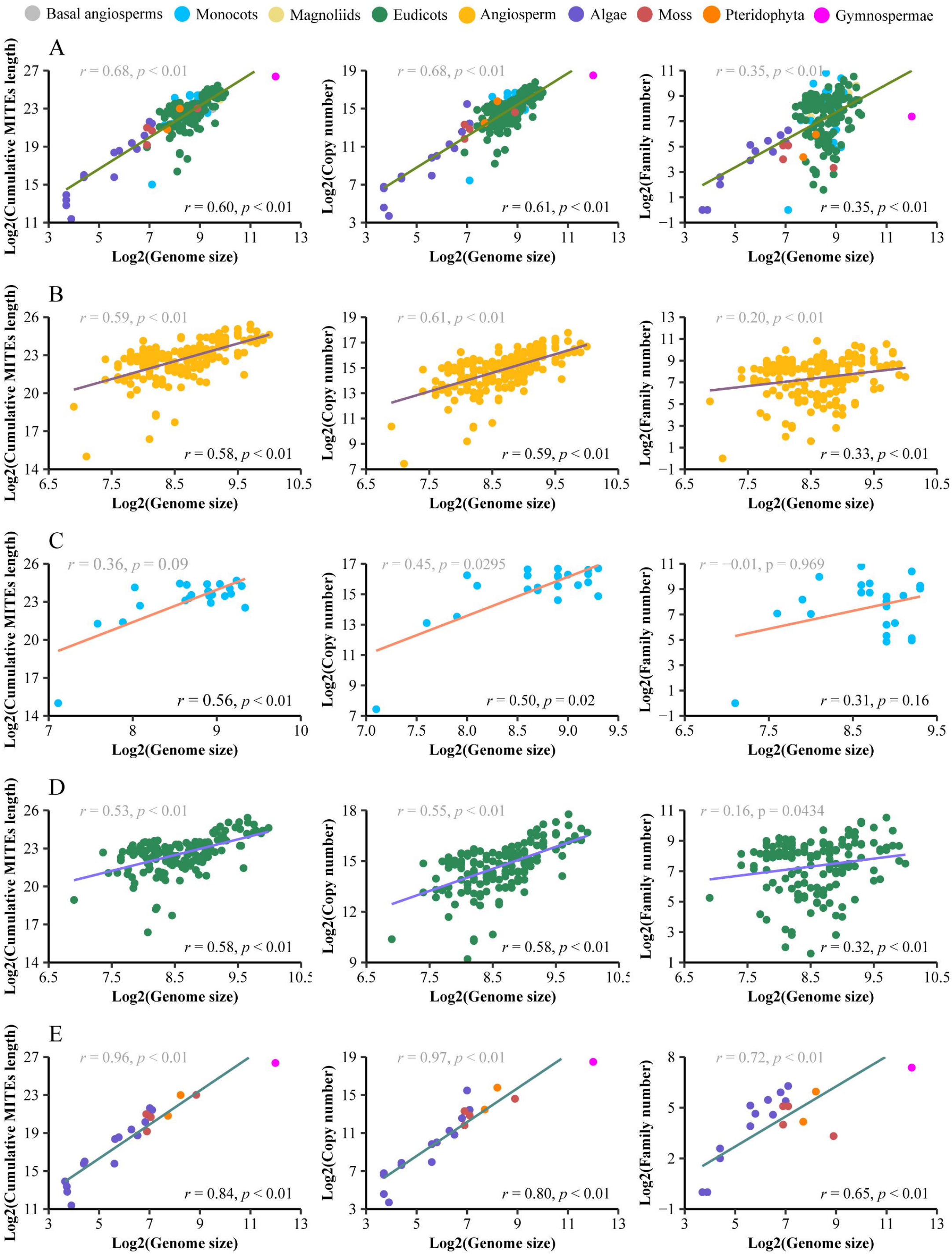

Figure S2 Correlation analysis between cumulative MITEs length, copy number, family number and genome size in different groups of plants. Spearman's correlation test was used to calculate correlation coefficients *r* and *p*-values. (A) All species. (B) Angiosperms. (C) Monocots. (D) Eudicots. (E) Species other than Angiosperms, i.e. Gymnospermae, Pteridophyta, Moss and Algae. The different

groups represented by different colors are marked in the legend. Values in light and dark shades indicate before and after phylogeny correction, respectively.

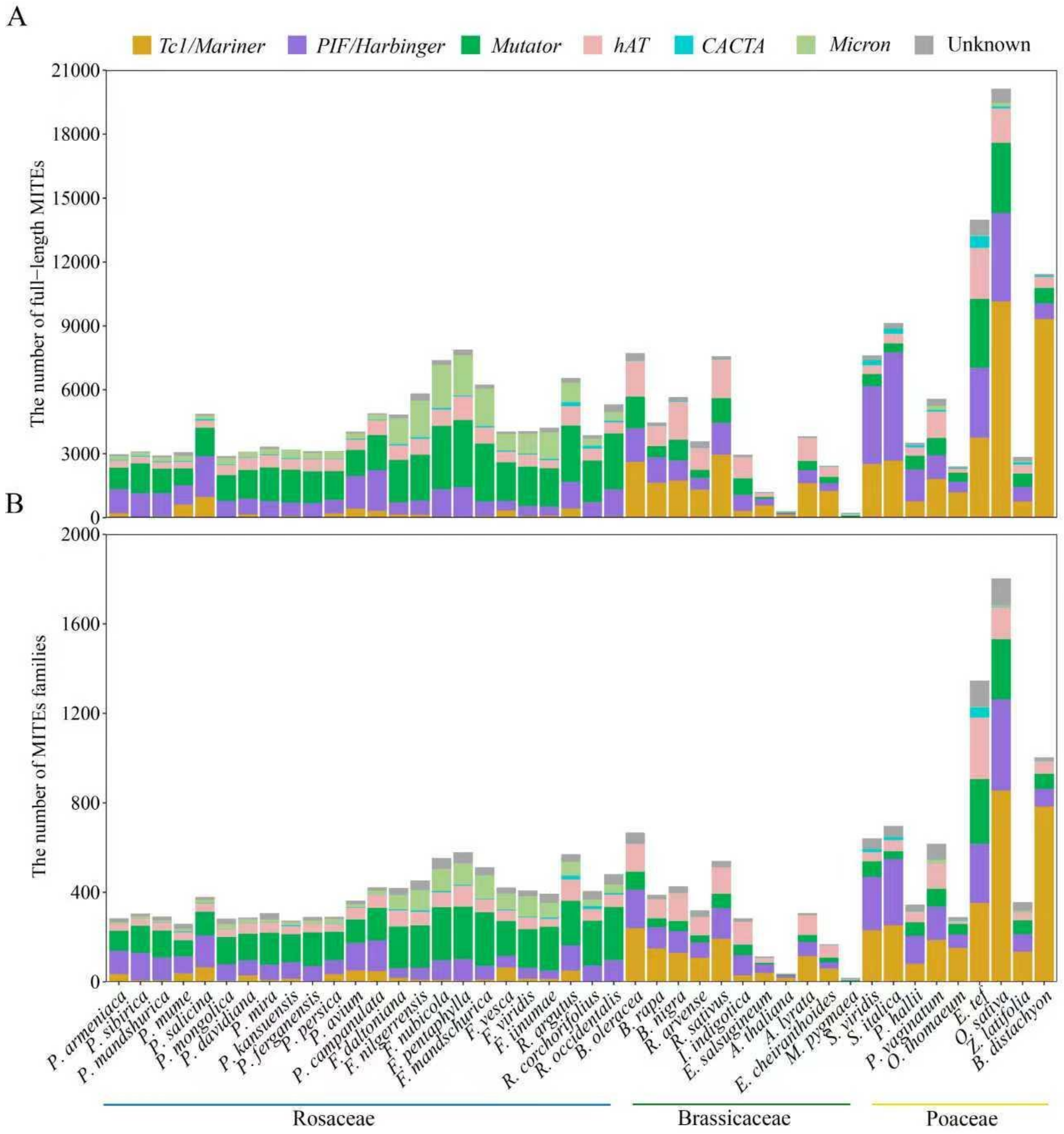

Figure S3 The number and family number of full-length MITEs in Rosaceae, Brassicaceae, and Poaceae. (A) Number of full-length MITEs. (B) The number of MITE families. The family to which the species belongs is underlined below, different colors represent different superfamilies.

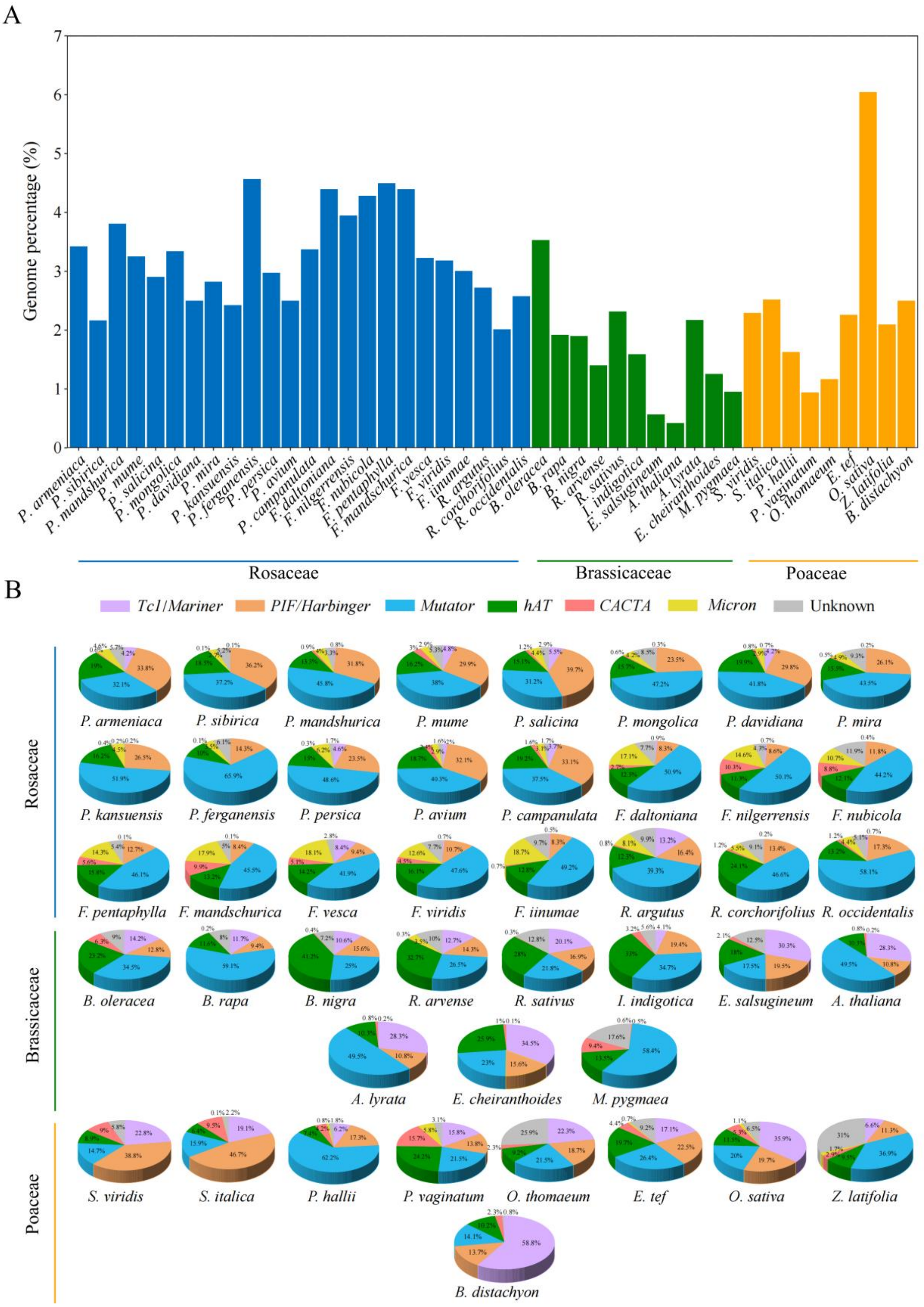

Figure S4 The content of MITEs in the whole genome of Rosaceae, Brassicaceae and Poaceae. (A) Genome proportion of MITEs. (B) The proportion of different superfamilies in MITEs in each species.

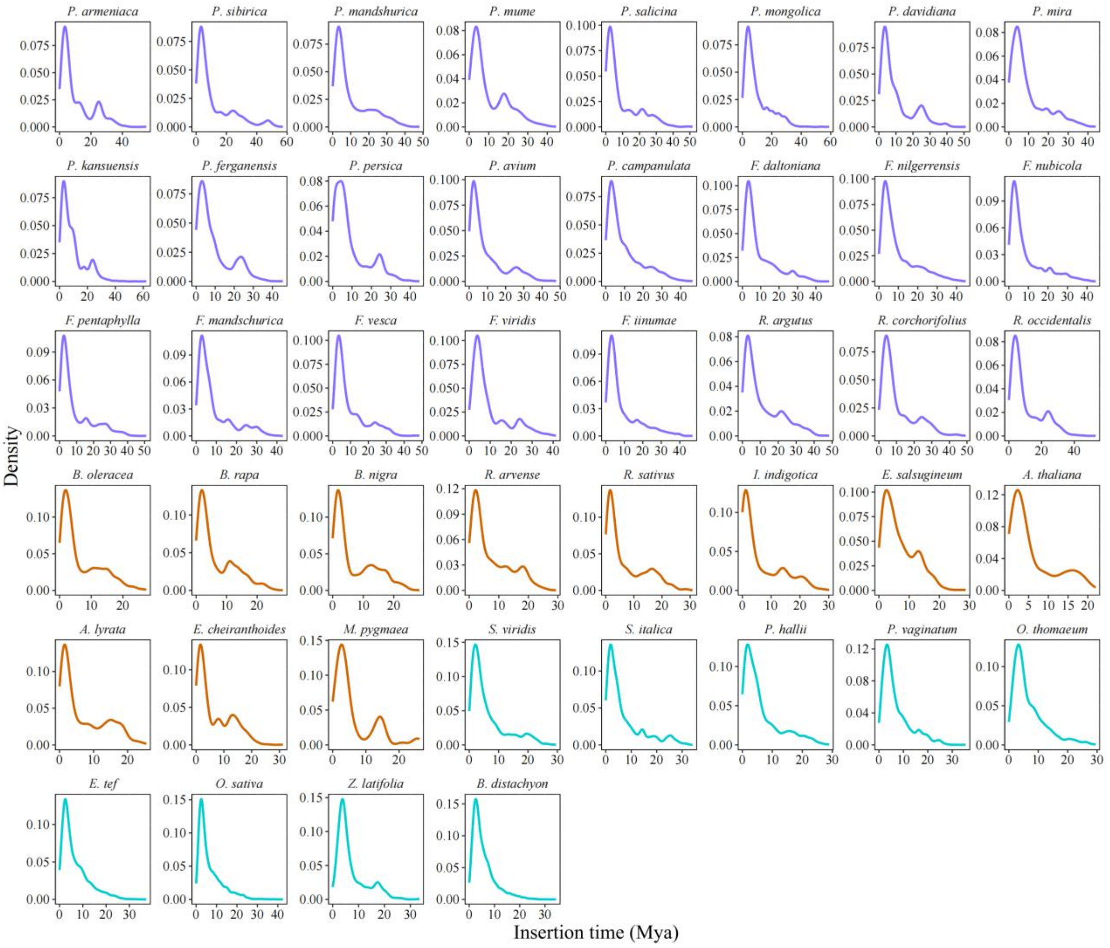

Figure S5 Insertion time of MITEs in the Rosaceae, Brassicaceae, and Poaceae genomes. Different families are represented by different colors.

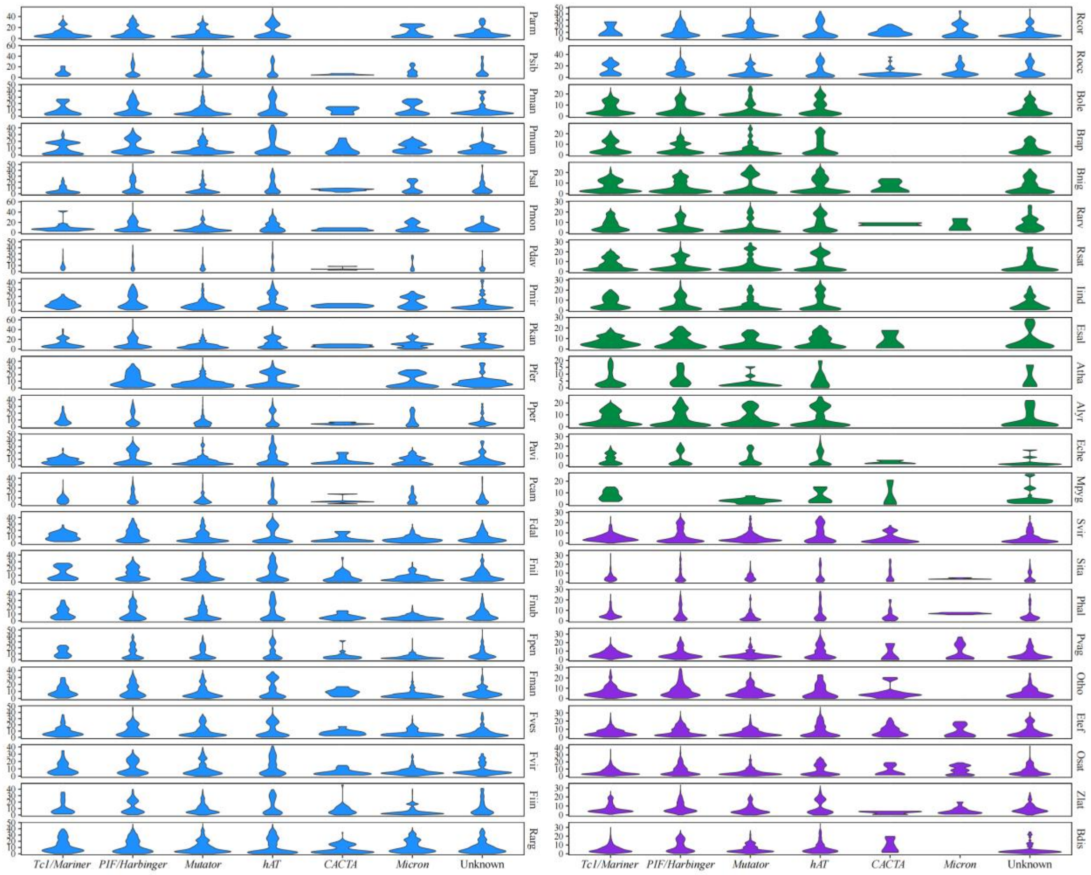

Figure S6 Insertion time of MITEs from different superfamilies in the Rosaceae, Brassicaceae, and Poaceae genomes. The same color of each violin diagram represents the same family. The parts on the right side of the violin plots are the abbreviation of every species name. For example, Atha represents *Arabidopsis thaliana*.

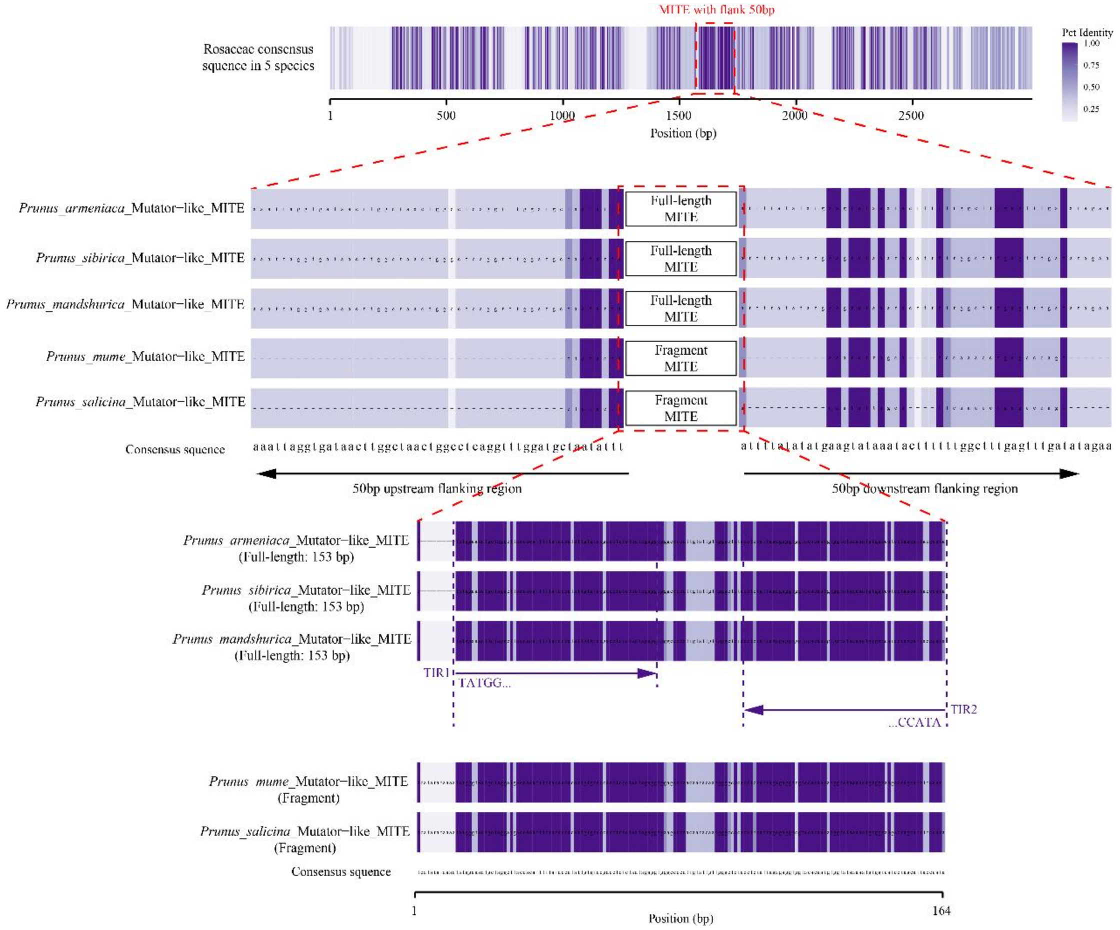

Figure S7 Sequence alignment of a shared orthologous MITE loci and flanking sequences in Rosaceae species.

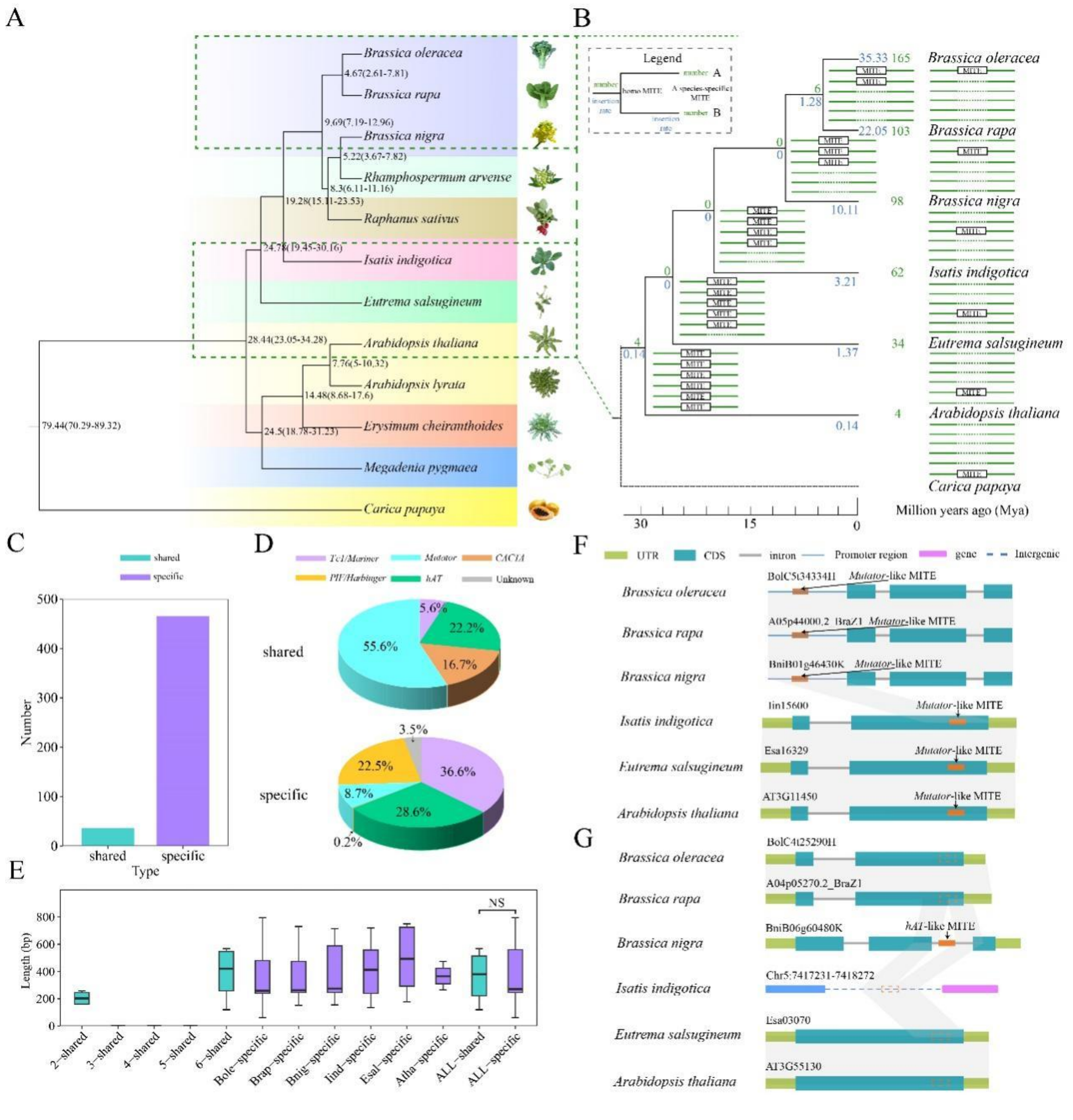

Figure S8 Evolution of MITEs during the differentiation of Brassicaceae species.

Same as Figure 3.

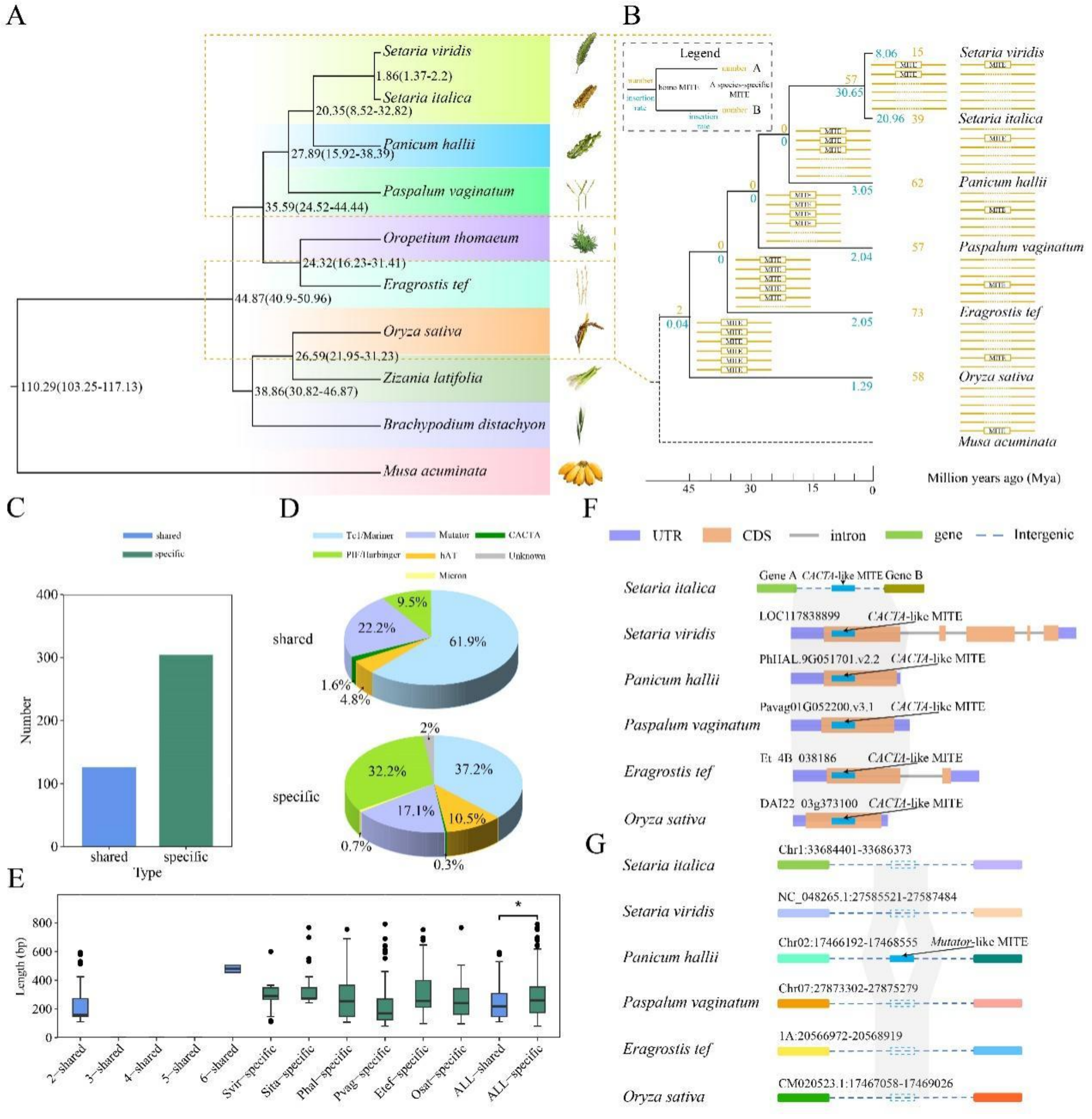

Figure S9 Evolution of MITEs during the differentiation of Poaceae species. Same as Figure 3.

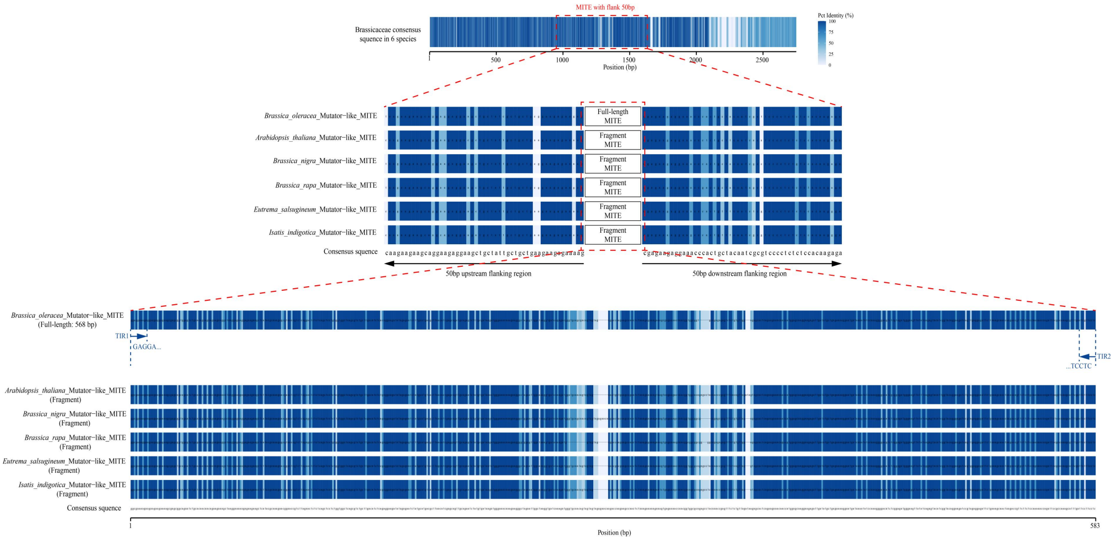

Figure S10 Sequence alignment of a shared orthologous MITE loci and flanking sequences in Brassicaceae species.

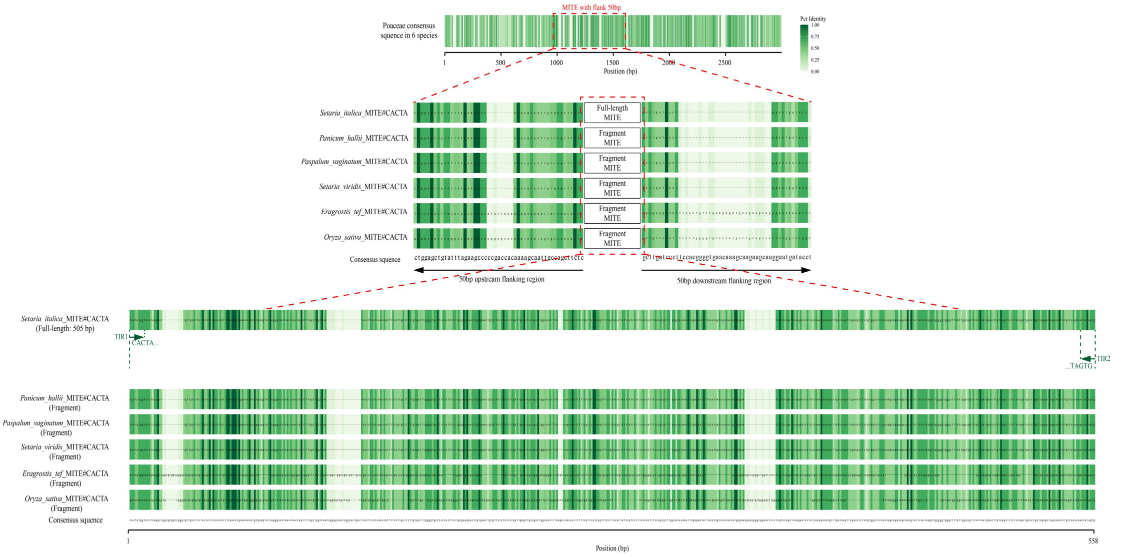

Figure S11 Sequence alignment of a shared orthologous MITE loci and flanking sequences in Poaceae species.

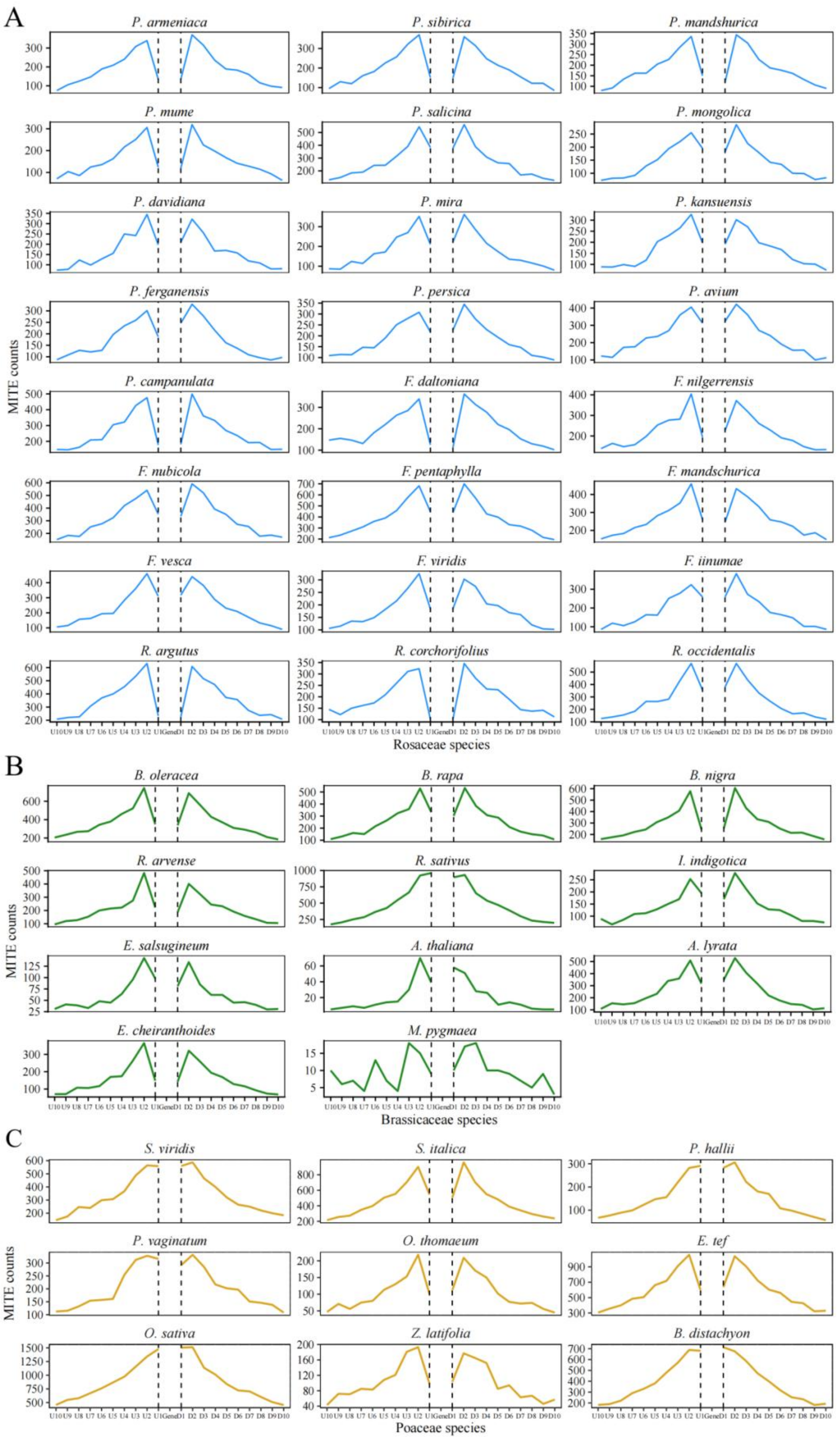

Figure S12 Distribution of MITEs in the flanking regions of genes. (A) Rosaceae. (B) Brassicaceae. (C) Poaceae.

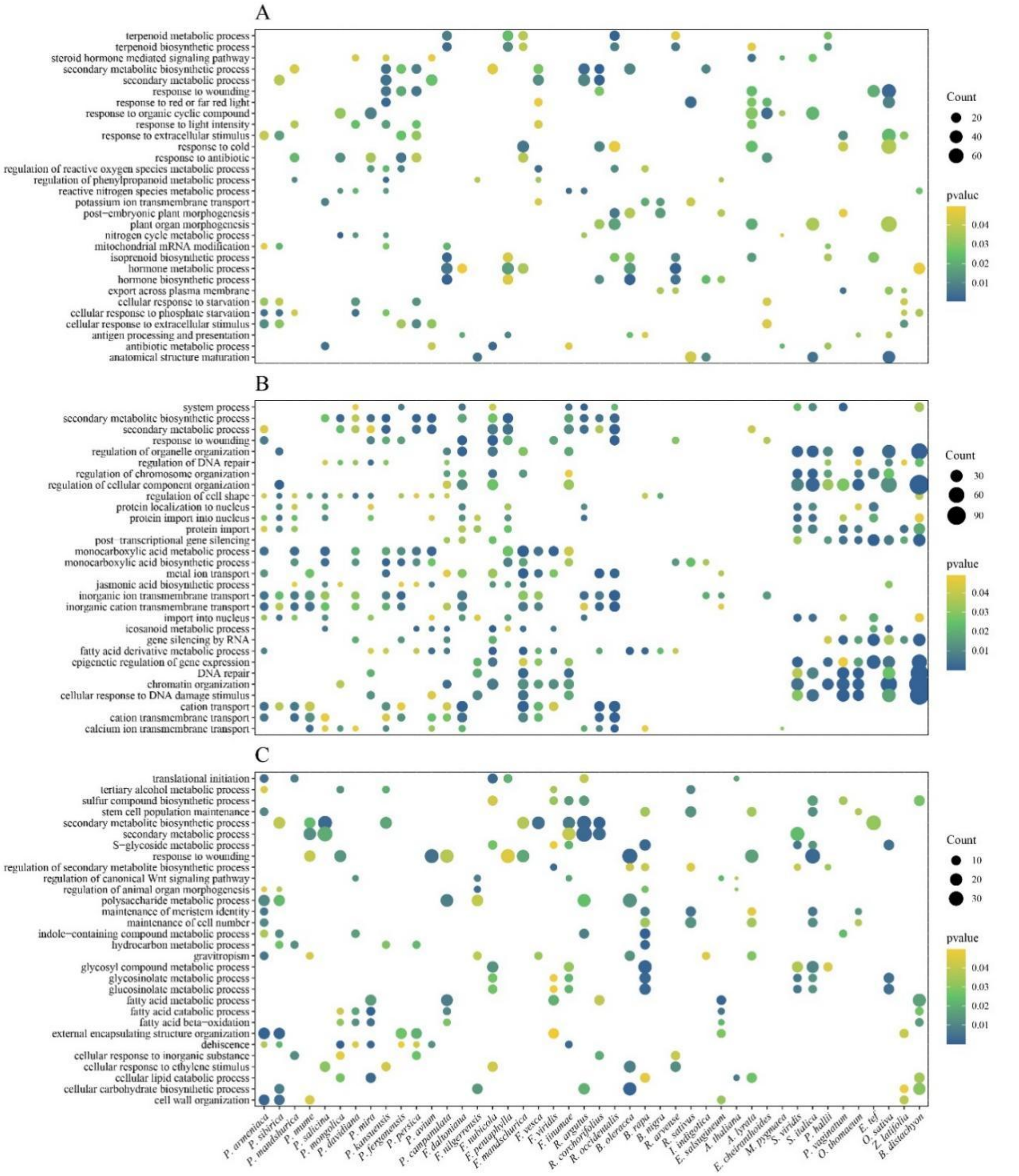

Figure S13 GO enrichment of genes associated with MITEs insertion in Rosaceae, Brassicaceae, and Poaceae species. (A) Function enrichment of genes with MITEs insertion in promoter. (B) Function enrichment of genes with MITEs insertion in intron. (C) Function enrichment of genes with MITEs insertion in downstream regions.

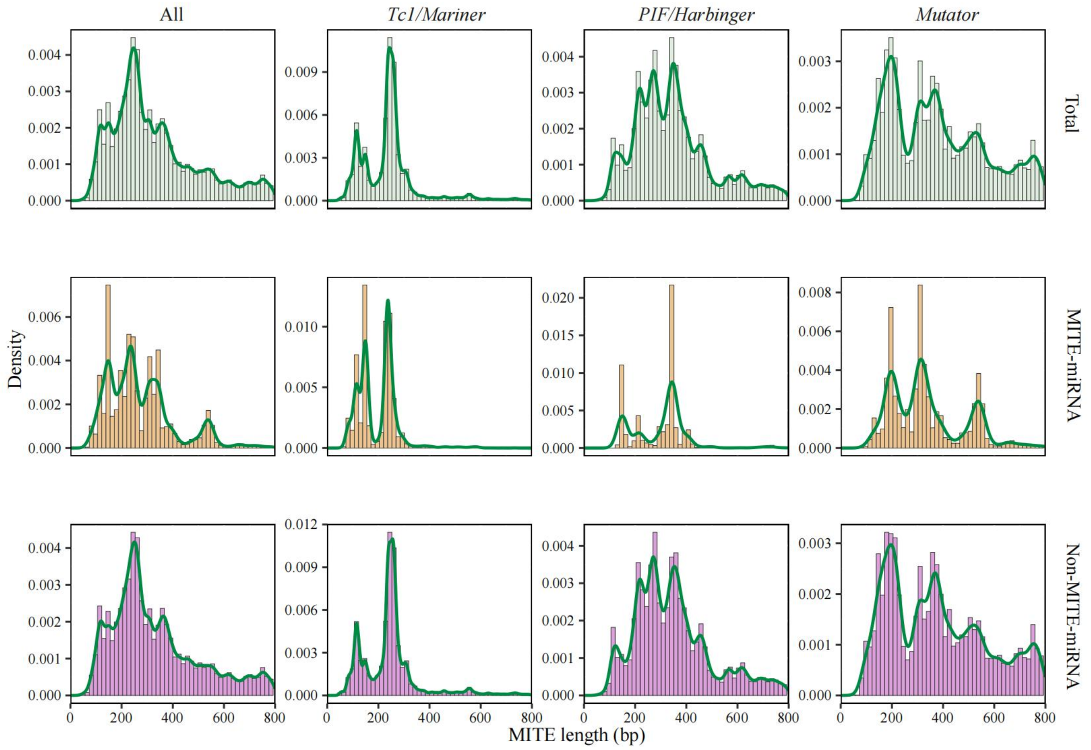

Figure S14 Comparisons of the length of MITE-miRNA and non-MITE-miRNA.

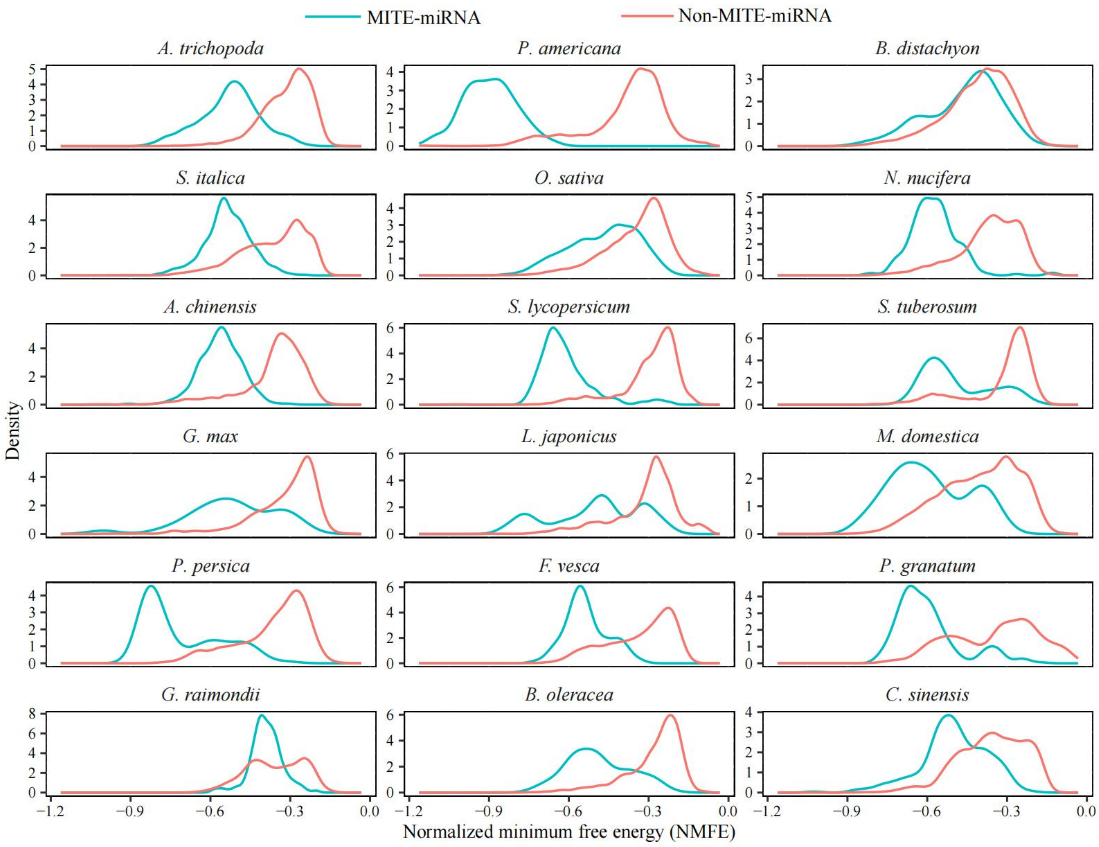

Figure S15 Comparisons of the NMFE of MITE-miRNA and non-MITE-miRNA.

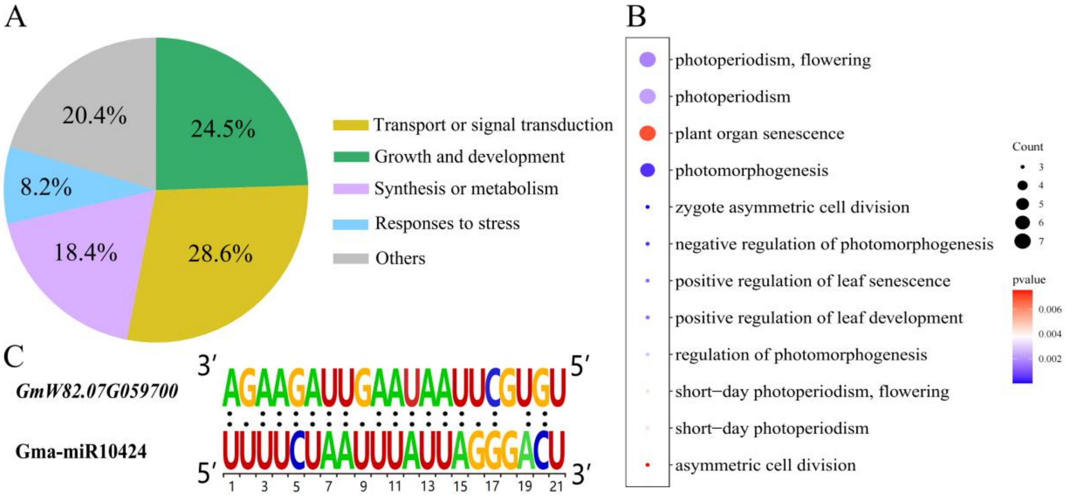

Figure S16 MITE-derived miRNA from soybean facilitated its growth and development. (A) Proportions of enriched GO terms associated with target genes of the MITE-derived miRNAs. (B) The base complementarity between Gma-miR10424 and its target site *GmW82.07G059700*. (C) Comparison of different types of enriched GO terms related to growth and development.

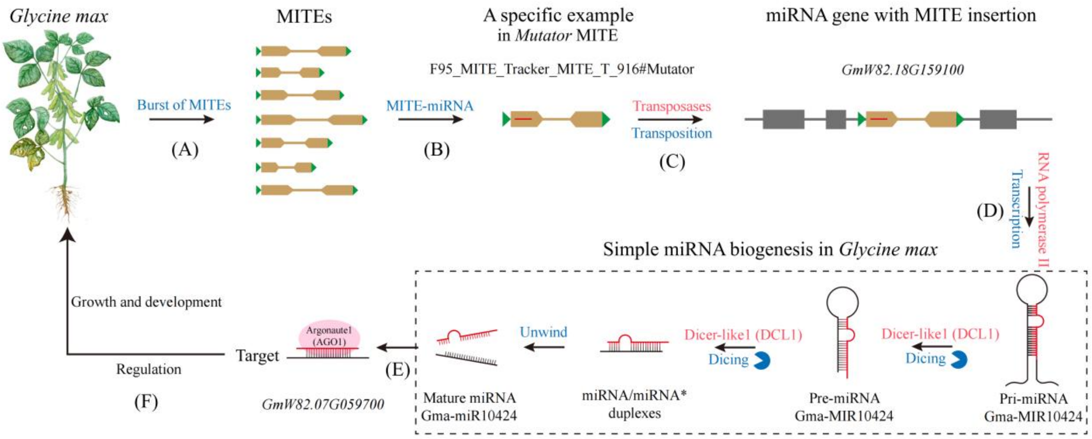

Figure S17 A model diagram for regulating the growth and development of soybean by F95_MITE_Tracker_MITE_T_916#*Mutator*. (A) Widespread bursts of MITEs in soybean. (B) MITE selectively generates MIR10424. (C) MIR10424 transposes with MITE. (D) The MITE hitchhikes host genes for transcription. (E) TIR regions of MITE fold into hairpin structures, which are recognized by the Dicer-like1 complex to generate novel miR10424. (F) miR10424 acts on target locus genes and regulates soybean growth and development.

Table S1 Summary of genome and MITE annotation information in 207 plant genomes

Species	Family	Contig N50 (Kb)	Scaffold N50 (Kb)	BUSCO (%)	LAI	Assembly Genome Size (Mb)	Database	MITE			
								Family number	Copy number	Total length (Mb)	Percentage in genome (%)
Amborella trichopoda	Amborellaceae	36,308	54,293	93.5	6.61	706	Phytozome	913	147422	35.00	4.96
Euryale ferox	Nymphaeaceae	4,750	24,897	93.4	6.39	725.2	CoGe	82	44999	8.10	1.12
Nymphaea colorata	Nymphaeaceae	2,136	25,516	94.4	14.4	409	NGDC	133	43832	5.96	1.46
Aristolochia contorta	Aristolochiaceae	2,320	30,380	90.28	11.82	209.27	CoGe	141	13097	2.23	1.07
Aristolochia fimbriata	Aristolochiaceae	5,157	12,891	96.8	14.9	258	NGDC	160	16774	4.45	1.72
Chimonanthus praecox	Calycanthaceae	8,130	63,910	95	8.42	737.03	NCBI	593	132011	28.82	3.91
Persea americana	Lauraceae	27,600	84,298	98.8	9.76	913	NCBI	900	77082	16.15	1.77
Acorus tatarinowii	Acoraceae	962	33,598	92.4	10.19	415.18	CNGB	423	39156	12.16	2.93
Acorus gramineus	Acoraceae	12,000	36,500	96.7	11.2	399.8	NGDC	424	76690	21.20	5.30
Spirodela polyrhiza	Araceae	3,339	7,689	92.8	12.47	138.49	CoGe	1	173	0.03	0.02
Zostera marina	Zosteraceae	7,000	34,600	95.7	10.35	260.5	Phytozome	132	77688	18.45	7.08
Acanthochlamys bracteata	Velloziaceae	7,550	8,620	97.7	12.46	191.61	NGDC	135	8821	2.53	1.32
Dioscorea alata	Dioscoreaceae	4,500	24,000	97.8	14.9	480	Phytozome	264	72931	14.43	3.01
Dioscorea zingiberensis	Dioscoreaceae	1,160	55,780	96.84	13.14	629	NCBI	529	106104	19.93	3.17
Brachypodium distachyon	Poaceae	22,000	30,200	99.3	10.77	271.9	NCBI	1005	48230	6.79	2.50
Panicum hallii	Poaceae	8,291	58,225	98.3	23.6	487.5	Phytozome	344	25045	7.92	1.63
Paspalum vaginatum	Poaceae	1,500	44,500	97.7	13.93	646.9	Phytozome	618	30011	6.08	0.94
Setaria italica	Poaceae	5,400	45,890	98.3	20.92	408.37	CNGB	702	44518	10.29	2.52
Setaria viridis	Poaceae	11,200	46,700	99	20.34	395.1	NCBI	641	40898	9.05	2.29
Eragrostis tef	Poaceae	1,550	27,140	98.1	20.24	576	CoGe	1348	56464	13.01	2.26

Oropetium thomaeum	Poaceae	2,020	20,500	98.9	20.64	236	CoGe	289	11772	2.75	1.16
Oryza sativa	Poaceae	1,400	30,300	99	20.12	377.6	NCBI	1804	101868	22.82	6.04
Zizania latifolia	Poaceae	4,480	32,790	97.71	14.84	547.38	NGDC	358	49682	11.48	2.10
Sparganium stoloniferum	Typhaceae	28,480	-	99.4	19.44	473	Figshare	199	75896	22.14	4.68
Ensete glaucum	Musaceae	10,840	55,000	96.8	8.17	481	Banana Genome Hub	40	47615	11.67	2.43
Musa acuminata	Musaceae	-	46,403	97.4	20.63	477	Figshare	73	104633	14.89	3.12
Musa balbisiana	Musaceae	1,830	41,415	91.3	10.67	492.77	Banana Genome Hub	29	74423	12.32	2.50
Musa beccarii	Musaceae	2,546	67,088	98.4	13.55	570	GigaDB	35	81989	17.44	3.06
Musa schizocarpa	Musaceae	6,493	44,786	92.3	13.56	525.28	Genoscope	80	79914	22.26	4.24
Musa troglodytarum	Musaceae	6,000	57,742	97.7	10.65	603	Banana Genome Hub	31	99616	26.87	4.46
Ceratophyllum demersum	Ceratophyllaceae	1,560	81,130	90.2	6.74	733.3	CoGe	469	162771	35.29	4.81
Corydalis tomentella	Papaveraceae	2,360	29,006	97.67	17.33	248.9	NGDC	440	43868	9.77	3.93
Nelumbo nucifera	Nelumbonaceae	5,100	108,283	94.24	10.44	807.01	CoGe	1478	142088	34.43	4.27
Santalum album	Santalaceae	10,640	26,510	96.5	30.87	229.59	NGDC	61	3304	0.96	0.42
Santalum yasi	Santalaceae	1,520	18,160	96.7	16.8	232.62	NGDC	76	5521	1.25	0.54
Taxillus chinensis	Loranthaceae	3,800	56,900	95	15.65	521.9	NCBI	139	69327	11.87	2.27
Fagopyrum tataricum	Polygonaceae	49,984	55,820	96.6	14.72	453	Figshare	281	59725	12.16	2.68
Camptotheca acuminata	Nyssaceae	1,470	18,280	96.4	11.5	414.95	Figshare	51	44714	6.07	1.46
Actinidia chinensis	Actinidiaceae	20,600	21,019	99.3	16.46	629	Provided by Dr. Junyang Yue	1151	95580	22.35	3.55
Coffea humblotiana	Rubiaceae	1,500	29,600	90.3	12.42	422	NCBI	444	27291	7.43	1.76
Gardenia jasminoides	Rubiaceae	1,000	44,000	95.8	12.39	535	NCBI	388	33861	8.93	1.67
Morinda officinalis	Rubiaceae	4,214	40,970	97.02	11.41	484.85	NCBI	341	39735	10.92	2.25
Ophiorrhiza pumila	Rubiaceae	18,490	40,060	97.1	20.14	439.9	NCBI	312	38309	8.92	2.03
Marsdenia tenacissima	Apocynaceae	6,570	-	91.11	12.66	381.76	CyVerse Data Store	252	42407	8.78	2.30
Catharanthus roseus	Apocynaceae	24,700	71,100	97.1	12.37	561.7	Figshare	167	33855	6.85	1.22
Sesamum indicum	Pedaliaceae	6,400	21,279	98.4	19.32	272.73	NCBI	92	10474	2.66	0.98

Lindernia brevidens	Linderniaceae	3,600	18,700	91	19.23	265	NCBI	252	28827	7.45	2.81
Callicarpa americana	Lamiaceae	7,511	29,054	93.8	14.69	506.11	GigaDB	444	31268	7.78	1.54
Scutellaria barbata	Lamiaceae	7,200	26,000	98.5	17.59	376.97	NCBI	278	40913	10.39	2.76
Tectona grandis	Lamiaceae	3,749	16,484	92.3	13.82	338	GigaDB	385	20600	4.42	1.31
Solanum arcanum	Solanaceae	10,900	69,600	98.3	16.23	862.4	NCBI	411	91784	31.08	3.60
Solanum galapagense	Solanaceae	12,320	-	98.5	13.74	859.93	NGDC	379	74403	20.56	2.39
Solanum habrochaites	Solanaceae	6,740	-	97.6	15.49	950.67	NGDC	406	76928	22.37	2.35
Solanum lycopersicum	Solanaceae	17,830	66,166	97.7	12.84	799.09	NGDC	348	64118	18.77	2.35
Solanum pimpinellifolium	Solanaceae	10,913	-	97.8	13.17	807.6	Sol Genomics Network	361	74579	17.59	2.18
Solanum tuberosum	Solanaceae	17,312	59,670	97.9	12.73	741.6	GigaDB	938	103744	24.17	3.26
Cissus rotundifolia	Vitaceae	186	27,600	92.4	15	350.69	NGDC	19	20586	5.77	1.64
Vitis adenoclada	Vitaceae	2,910	25,260	90.64	16.25	498.27	NGDC	126	20632	5.15	1.03
Vitis amurensis	Vitaceae	282	26,085	94.6	13.52	604.56	NGDC	161	33216	7.50	1.24
Vitis arizonica	Vitaceae	-	25,900	96.4	17.76	503	Zenodo	118	21773	4.99	0.99
Vitis vinifera	Vitaceae	26,890	26,899	98.5	18.58	494.87	Zenodo	157	29063	5.94	1.20
Vitis labrusca	Vitaceae	2,500	-	97.1	17.77	502	Figshare	145	22390	5.74	1.14
Abrus cantoniensis	Fabaceae	18,930	18,950	97.1	14.78	381.27	NGDC	31	6158	8.99	2.36
Amphicarpaea edgeworthii	Fabaceae	1,440	28,470	93.4	7.67	343.78	Figshare	75	14096	3.62	1.05
Astragalus sinicus	Fabaceae	1,500	78,420	91.1	12.21	595.52	Provided by Dr. Weidong C	614	121247	27.94	4.69
Glycine max	Fabaceae	32,000	-	99.8	9.75	933	NGDC	205	124827	19.10	2.05
Glycine soja	Fabaceae	3,300	50,700	99.7	8.4	1013.2	NCBI	181	105599	25.77	2.54
Lablab purpureus	Fabaceae	11,000	38,100	98.5	21.9	426.2	e!DAL	112	19631	6.54	1.53
Lotus japonicus	Fabaceae	807	95,600	98.2	19.1	554	CoGe	844	79071	19.15	3.46
Lupinus angustifolius	Fabaceae	5,650	30,795	94.4	10.31	615.8	CNGB	439	59699	12.81	2.08
Phaseolus acutifolius	Fabaceae	6,178	45,200	97.4	22.6	512.6	Phytozome	167	45233	10.13	1.98
Phaseolus vulgaris	Fabaceae	19,800	54,900	99.2	15.78	566.2	NCBI	181	76330	23.07	4.08

Vigna mungo	Fabaceae	350	42,880	94.17	14.6	454.4	NCBI	220	48417	8.91	1.96
Vigna radiata	Fabaceae	10,340	43,790	98.02	15.47	475.35	Figshare	327	61840	11.97	2.52
Vigna umbellata	Fabaceae	7,500	53,400	97.2	16.22	626.9	NCBI	237	73244	13.58	2.17
Vigna unguiculata	Fabaceae	24,190	49,110	96.28	13.69	550.31	NCBI	274	104688	20.27	3.68
Bauhinia variegata	Fabaceae	4,550	22,089	98.7	18.37	326.4	NCBI	41	9402	1.47	0.45
Cercis chinensis	Fabaceae	33,270	47,428	98.3	21.05	352.84	NGDC	231	15647	3.33	0.95
Entada phaseoloides	Fabaceae	6,340	30,900	98.8	7.62	456.18	CNGB	62	10936	2.58	0.56
Malus domestica	Rosaceae	2,317	23,924	97.9	14.97	652.4	http://bioinfo.bti.cornell.edu/	1226	148382	34.76	5.33
Prunus armeniaca	Rosaceae	1,800	25,150	97.3	18.23	203.93	GDR	284	32400	6.98	3.42
Prunus avium	Rosaceae	3,247	42,624	97.4	12.66	344.29	GDR	363	41159	8.60	2.50
Prunus campanulata	Rosaceae	18,310	32,687	96.81	17.96	280.2	http://tree-bio.hzau.edu.cn/	422	44821	9.44	3.37
Prunus davidiana	Rosaceae	22,637	28,549	97.1	12.56	259.27	GDR	287	28373	6.48	2.50
Prunus dulcis	Rosaceae	1,748	30,643	97.1	20.99	164.55	NCBI	282	29771	6.71	4.08
Prunus humilis	Rosaceae	1,450	26,230	98.3	10.68	229.21	Figshare	230	24864	5.84	2.55
Prunus kansuensis	Rosaceae	24,419	28,885	95.6	18.83	253.17	GDR	273	32782	6.14	2.42
Prunus mandshurica	Rosaceae	3,200	29,440	94.1	15.49	223.66	GDR	292	30344	8.52	3.81
Prunus mira	Rosaceae	25,948	27,933	97.1	14.39	253.63	GDR	307	30831	7.16	2.82
Prunus mume	Rosaceae	2,750	29,400	96.4	14.28	237.8	GDR	260	32202	7.73	3.25
Prunus persica	Rosaceae	6,866	27,890	98	21.02	236.53	GDR	292	29035	7.03	2.97
Prunus salicina	Rosaceae	1,370	34,457	98.64	15.71	282.38	GDR	379	41843	8.19	2.90
Prunus sibirica	Rosaceae	1,700	33,800	98.2	13.84	259.43	GDR	279	24325	5.61	2.16
Prunus ferganensis	Rosaceae	26,470	28,247	97.2	15.91	261.3	GDR	290	31816	11.92	4.56
Prunus mongolica	Rosaceae	24,330	26,540	98.76	22.66	233.16	NGDC	285	31980	7.79	3.34
Prunus tenella	Rosaceae	18,100	25,637	94.7	19.63	231	NGDC	239	28290	6.01	2.60
Fragaria daltoniana	Rosaceae	4,290	34,975	93.3	7.59	288.97	GDR	419	53803	12.69	4.39
Fragaria iinumae	Rosaceae	10,670	33,980	94.8	17.3	240.58	GDR	393	33631	7.22	3.00

Fragaria mandschurica	Rosaceae	1,290	29,635	90.7	16.04	228	GDR	512	38416	10.03	4.40
Fragaria nilgerrensis	Rosaceae	3,160	34,281	94.5	10.65	288.43	GDR	453	45197	11.38	3.95
Fragaria nubicola	Rosaceae	2,600	35,000	87.5	17.62	273	GDR	554	44834	11.68	4.28
Fragaria pentaphylla	Rosaceae	32,376	34,602	98.76	23.23	256.74	Figshare	580	45268	11.54	4.50
Fragaria vesca	Rosaceae	34,000	-	96.1	16.36	220	GDR	421	30031	7.09	3.22
Fragaria viridis	Rosaceae	9,830	31,444	94.5	15.98	223.08	GDR	408	29746	7.09	3.18
Rubus argutus	Rosaceae	650	38,600	91.7	17.6	298.24	GDR	546	31754	8.12	2.72
Rubus corchorifolius	Rosaceae	2,490	29,500	94.7	24.36	330.25	NGDC	406	23994	6.65	2.01
Rubus occidentalis	Rosaceae	5,100	41,100	98.7	15.26	290	GDR	481	30108	7.46	2.57
Artocarpus nanchuanensis	Moraceae	2,090	25,150	98.08	8.07	769.44	GigaDB	89	17726	2.87	0.37
Morus notabilis	Moraceae	75,380	-	98.51	15.21	410.45	NGDC	831	53541	11.47	2.79
Ziziphus jujuba	Rhamnaceae	32,990	-	98.5	18.24	393.3	NGDC	61	10939	2.99	0.76
Boehmeria nivea	Urticaceae	10,510	19,550	96.9	16.96	270.21	NCBI	267	40710	11.32	4.19
Carya illinoinensis	Juglandaceae	26,500	26,500	99.1	19.61	636.26	NCBI	429	68696	12.82	2.01
Juglans regia	Juglandaceae	1,083	37,114	95.1	12.73	573.9	GigaDB	767	92841	20.61	3.59
Ostrya rehderiana	Betulaceae	-	40,927	98.7	11.66	366.2	Figshare	482	46319	10.42	2.84
Casuarina cunninghamiana	Casuarinaceae	2,232	33,000	97.9	20.57	293.48	NCBI	7	1238	0.30	0.10
Casuarina equisetifolia	Casuarinaceae	5,391	29,200	98	21.51	268.94	NCBI	4	589	0.09	0.03
Casuarina glauca	Casuarinaceae	1,118	32,800	97.9	20.54	296.63	NCBI	8	1328	0.33	0.11
Citrullus lanatus	Cucurbitaceae	32,500	-	99.13	9.47	369.3	NGDC	60	32592	5.64	1.53
Cucumis melo	Cucurbitaceae	2,860	30,500	92.78	11.74	386	NCBI	45	29628	3.49	0.90
Cucumis metuliferus	Cucurbitaceae	2,900	-	93.54	10.76	323.3	NGDC	35	12735	2.14	0.66
Cucumis sativus	Cucurbitaceae	8,900	31,100	91.3	10.05	226.2	GigaDB	14	11228	1.74	0.77
Herpetospermum pedunculosum	Cucurbitaceae	24,390	71,400	97.65	20.47	804.11	NCBI	338	224286	45.06	5.60
Lagenaria siceraria	Cucurbitaceae	11,200	28,400	95.5	11.32	297	NCBI	234	51659	8.62	2.90
Averrhoa carambola	Oxalidaceae	4,220	32,669	96.3	16.33	320	NGDC	42	7642	1.88	0.59

Populus alba	Salicaceae	5,430	24,761	98.5	3.99	405.31	Figshare	43	11314	2.75	0.68
Populus deltoides	Salicaceae	2,620	21,500	98.2	14.71	429.3	NCBI	24	10929	3.23	0.75
Populus euphratica	Salicaceae	2,039	23,893	92.5	20.85	511.25	NGDC	25	9154	1.89	0.37
Populus simonii	Salicaceae	1,940	19,598	97.9	13.11	441.38	NCBI	30	13437	4.18	0.95
Populus koreana	Salicaceae	6,410	19,875	97.8	12.68	401.4	NGDC	30	15295	4.34	1.08
Populus wilsonii	Salicaceae	16,300	22,000	98.57	8.15	477.35	Figshare	70	47143	10.59	2.22
Salix arbutifolia	Salicaceae	2,751	15,423	97.4	12.59	324.9	NGDC	70	49343	9.14	2.81
Salix brachista	Salicaceae	9,522	17,922	96.1	16.8	339.58	NGDC	109	35107	7.31	2.15
Salix chaenomeloides	Salicaceae	6,783	17,154	97.8	14.2	335.1	NGDC	38	18676	4.47	1.33
Salix dunnii	Salicaceae	16,657	17,280	96.6	13.48	328	NCBI	40	23582	4.16	1.27
Triadica sebifera	Euphorbiaceae	29,398	-	99	13.27	739	NCBI	83	52653	9.71	1.31
Manihot esculenta	Euphorbiaceae	26,000	34,200	98.8	19.23	703.7	NCBI	133	96667	26.53	3.77
Ricinus communis	Euphorbiaceae	8,960	31,930	95.63	9.55	316	NGDC	46	5316	1.69	0.53
Psidium guajava	Myrtaceae	15,800	40,400	95.7	14.29	443.8	NCBI	151	26714	6.90	1.56
Rhodomyrtus tomentosa	Myrtaceae	43,800	43,800	97.7	16.16	470.35	NCBI	127	14238	1.99	0.42
Syzygium aromaticum	Myrtaceae	3,800	35,418	98.2	13.79	370	NCBI	168	15563	3.76	1.02
Melastoma dodecandrum	Melastomataceae	3,000	21,830	96.07	21.04	299.81	NGDC	58	16375	12.94	4.31
Melastoma candidum	Melastomataceae	2,018	20,500	92.9	24.55	256.2	NCBI	55	8904	2.24	0.88
Punica granatum	Lythraceae	4,490	39,960	93.33	9.41	320.31	NCBI	300	28850	7.02	2.19
Trapa natans	Lythraceae	13,515	21,554	97.7	6.31	489.6	NGDC	7	37400	3.95	0.81
Trapa incisa	Lythraceae	13,768	21,690	97.6	6.58	472.74	NGDC	17	21497	4.11	0.87
Gossypioides kirkii	Malvaceae	9,920	42,970	95	9.86	538	NCBI	155	26428	7.30	1.36
Gossypium raimondii	Malvaceae	17,044	57,717	95.4	11.06	750	Figshare	322	40612	9.69	1.29
Theobroma cacao	Malvaceae	16,000	39,500	98.5	12.22	382.4	NCBI	30	5525	1.44	0.38
Dipterocarpus turbinatus	Dipterocarpaceae	245	29,439	90.7	14.14	421.17	CNGB	482	40315	10.20	2.42
Hopea hainanensis	Dipterocarpaceae	6,614	69,706	91.4	17.13	434.31	CNGB	434	36525	8.81	2.03

Hopea mollissima	Dipterocarpaceae	2,800	29,300	98	18.35	361.6	CNGB	260	18488	4.59	1.27
Shorea henryana	Dipterocarpaceae	10,600	41,800	98.3	14.49	302.6	CNGB	113	8670	2.39	0.79
Shorea wangtianshuea	Dipterocarpaceae	9,600	39,700	98.4	15.15	315	CNGB	135	8508	2.25	0.71
Vatica rassak	Dipterocarpaceae	2,100	7,500	97.7	20.12	476.2	CNGB	116	15964	4.18	0.88
Carica papaya	Caricaceae	11,500	36,982	97.5	7.05	351	NGDC	3	1612	0.21	0.06
Capparis spinosa	Capparaceae	9,360	15,150	96.8	17.19	274.53	GigaDB	9	9641	2.81	1.03
Arabidopsis arenosa	Brassicaceae	4,900	19,700	95.9	22.5	269.15	NCBI	283	12864	3.64	1.35
Arabidopsis lyrata	Brassicaceae	-	-	99	25.27	200	EnsemblGenomes	307	15946	4.34	2.17
Arabidopsis suecica	Brassicaceae	5,000	19,800	99.2	31.99	272.39	NCBI	254	12425	3.51	1.29
Arabidopsis thaliana	Brassicaceae	11,195	23,460	99.3	17.42	119.67	NCBI	38	1330	0.50	0.42
Brassica juncea	Brassicaceae	56,272	58,583	99.7	15.6	884.8	NCBI	814	79479	21.89	2.47
Brassica nigra	Brassicaceae	15,656	61,872	95.6	14.95	500.55	Figshare	428	33109	9.52	1.90
Brassica oleracea	Brassicaceae	9,491	58,258	95.8	16.97	554.98	Genoscope	667	71651	19.60	3.53
Brassica rapa	Brassicaceae	10,256	39,217	96.6	10.02	443.95	Genoscope	389	34098	8.52	1.92
Camelina hispida	Brassicaceae	1,208	41,586	96	25.47	283.17	NCBI	285	19219	5.32	1.88
Camelina laxa	Brassicaceae	589	31,824	96	20.8	199.99	NCBI	164	10125	5.15	2.58
Camelina neglecta	Brassicaceae	6,834	29,060	97.6	21.03	192.5	NCBI	141	7383	2.21	1.15
Erysimum cheiranthoides	Brassicaceae	-	22,400	99	25.14	174.5	Erysimum Database	168	9067	2.19	1.26
Eutrema salsugineum	Brassicaceae	3,053	36,820	99.1	8.79	295.5	NCBI	113	6153	1.68	0.57
Isatis indigotica	Brassicaceae	1,176	36,160	98.33	23.52	293.88	Figshare	286	15128	4.67	1.59
Megadenia pygmaea	Brassicaceae	1,810	34,800	98.9	9.34	215.2	NGDC	18	9849	2.05	0.95
Raphanus sativus	Brassicaceae	1,248	53,944	94.1	21.51	504.5	Plant GARDEN	539	49864	11.68	2.31
Rhamphospermum arvense	Brassicaceae	7,439	31,689	96.3	-	416.11	Figshare	320	21652	5.82	1.40
Thlaspi arvense	Brassicaceae	19,463	64,900	98.7	16.06	526	ENA	118	21963	11.08	2.11
Atalantia buxifolia	Rutaceae	4,200	32,200	98.2	19.51	304.2	NCBI	278	20654	6.48	2.13
Citrus australis	Rutaceae	-	36,300	98.8	19.41	331.1	NGDC	305	16796	5.42	1.64

Citrus sinensis	Rutaceae	32,900	32,900	99.2	20.97	299	NCBI	370	22255	7.02	2.35
Citrus trifoliata	Rutaceae	843	27,700	97.2	16.76	264.9	Phytozome	265	15196	4.94	1.87
Murraya paniculata	Rutaceae	18,250	23,920	98.24	14.16	216.87	NGDC	136	8341	2.66	1.23
Toona ciliata	Meliaceae	4,480	17,615	94.44	11.48	520.64	CNGB	16	15258	5.28	1.01
Toona sinensis	Meliaceae	1,500	21,500	97.5	15.49	596	CNGB	35	39948	14.26	2.39
Pistacia vera	Anacardiaceae	680	35,600	98.6	15.2	614	NCBI	446	69835	16.89	2.75
Aesculus wilsonii	Sapindaceae	3,750	28,020	91	11.03	579.01	NGDC	468	62318	13.29	2.29
Dimocarpus longan	Sapindaceae	12,100	29,500	98.1	20.76	455.5	NGDC	140	20938	5.84	1.28
Litchi chinensis	Sapindaceae	752	30,902	96.2	19.46	470	Mendeley Data	180	20936	5.26	1.12
Nephelium lappaceum	Sapindaceae	1,300	21,650	97.5	11.99	409.3	https://bcb.unl.edu/	54	17466	4.15	1.01
Gracilaria domingensis	Gracilariaceae	133	189	-	-	77.74	NCBI	44	2425	0.68	0.87
Gracilariopsis chorda	Gracilariaceae	220	-	87.6	-	92.18	NCBI	24	1811	0.44	0.48
Neoporphyra haitanensis	Bangiaceae	650	7,796	80.2	-	49.67	NCBI	35	912	0.34	0.68
Ostreococcus tauri	Bathycoccaceae	48	770	-	-	12.56	NCBI	1	97	0.02	0.12
Ostreococcus lucimarinus	Bathycoccaceae	709	709	-	-	13.2	NCBI	1	110	0.01	0.08
Bathycoccus prasinos	Bathycoccaceae	663	956	-	-	14.96	NCBI	1	13	0.00	0.02
Micromonas commoda	Mamiellaceae	1,394	1,394	-	-	20.99	NCBI	4	233	0.06	0.27
Pedinomonas minor	Pedinomonadaceae	2,058	-	91	-	54.96	CNGB	25	1036	0.39	0.70
Coccomyxa subellipsoidea	-	1,960	-	-	-	48.8	NCBI	15	247	0.06	0.12
Picochlorum costavermella	-	244	764	-	-	13.33	NCBI	1	24	0.01	0.05
Nannochloris desiccata	Chlorellaceae	1,642	-	97.5	-	21.55	NCBI	6	198	0.07	0.31
Chlamydomonas incerta	Chlamydomonadaceae	1,580	-	96.5	-	129.24	https://datashare.ed.ac.uk/	42	45716	3.23	2.50
Chlamydomonas reinhardtii	Chlamydomonadaceae	6,949	6,949	99.74	-	113.9	NCBI	60	6035	1.18	1.03
Volvox carteri	Volvocaceae	44	1,492	-	-	137.68	NCBI	78	11106	2.82	2.05
Physcomitrella patens	Funariaceae	465	2,800	98.7	-	462.3	CoGe	10	24987	8.49	1.84
Anthoceros angustus	Anthocerotaceae	797	1,092	85.04	-	119.33	DRYAD	16	3602	0.59	0.50

Anthoceros agrestis	Anthocerotaceae	156	17,300	91.4	-	116.9	Hornworts	34	10174	2.08	1.78
Anthoceros punctatus	Anthocerotaceae	1,700	-	95.6	-	132.8	Hornworts	34	7422	1.68	1.26
Selaginella moellendorffii	Selaginellaceae	120	1,750	-	-	212.6	NCBI	18	11342	1.86	0.87
Selaginella tamariscina	Selaginellaceae	201	408	-	-	300.73	NCBI	62	56235	8.38	2.79
Gnetum montanum	Gnetaceae	25	475	81	-	4070	DRYAD	166	368869	87.12	2.14

Table S2 The information of RNA sequence reads

Species	BioProject	SRA	Tissue
Paspalum vaginatum	PRJNA746310	SRR15558341	Root
		SRR15558343	Root
		SRR15558344	Root
Brachypodium	PRJDB13671	DRR378316	Leaf
distachyon		DRR378315	Leaf
		DRR378317	Leaf
Prunus salicina	PRJNA645255	SRR12234096	Flower buds
		SRR12234097	Flower buds
		SRR12234098	Flower buds
Malus domestica	PRJNA591623	SRR12030790	Fruit
		SRR12030789	Fruit
		SRR12030788	Fruit
Arabidopsis thaliana	PRJDB17878	DRR545898	Ovule
		DRR545897	Ovule
		DRR545896	Ovule
Eutrema salsugineum	PRJEB113410625	ERR11403365	Leaf
		ERR11403361	Leaf
		ERR11403366	Leaf

Table S3 Number of miRNAs retrieved from PmiREN and miRBase databases

Species	Total miRNA number	Only in PmiREN	Only in miRBase	Both in PmiREN and mirBase
Chlamydomonas reinhardtii	174	124	17	33
Volvox carteri	200	200	0	0
Physcomitrella patens	306	59	48	199
Selaginella moellendorffii	161	103	26	32
Amborella trichopoda	236	112	50	74
Persea americana	92	92	0	0
Spirodela polyrhiza	55	55	0	0
Zostera marina	68	68	0	0
Brachypodium distachyon	507	190	101	216
Panicum hallii	93	93	0	0
Setaria italica	190	190	0	0
Eragrostis tef	152	152	0	0
Oryza sativa	954	350	259	345
Musa acuminata	274	274	0	0
Nelumbo nucifera	143	143	0	0
Actinidia chinensis	182	182	0	0
Catharanthus roseus	65	65	0	0
Solanum habrochaites	112	112	0	0
Solanum lycopersicum	356	244	32	80
Solanum pimpinellifolium	133	133	0	0
Solanum tuberosum	286	62	117	107
Vitis vinifera	315	152	26	137
Glycine max	964	280	240	444
Glycine soja	416	403	4	9
Lotus japonicus	612	313	153	146

Phaseolus vulgaris	135	127	3	5
Vigna radiata	108	108	0	0
Vigna unguiculata	147	129	8	10
Malus domestica	432	124	165	143
Prunus avium	178	178	0	0
Prunus dulcis	152	152	0	0
Prunus persica	344	164	51	129
Fragaria vesca	199	81	13	105
Ziziphus jujuba	152	152	0	0
Juglans regia	204	204	0	0
Citrullus lanatus	139	139	0	0
Cucumis melo	143	23	34	86
Cucumis sativus	16	11	5	0
Lagenaria siceraria	60	60	0	0
Populus euphratica	178	174	4	0
Manihot esculenta	288	114	34	140
Ricinus communis	124	61	14	49
Punica granatum	33	33	0	0
Gossypium raimondii	296	0	296	0
Theobroma cacao	122	40	52	30
Carica papaya	122	43	25	54
Arabidopsis lyrate	275	70	58	147
Arabidopsis thaliana	363	37	142	184
Brassica juncea	366	366	0	0
Brassica nigra	138	138	0	0
Brassica oleracea	73	63	5	5
Brassica rapa	320	224	30	66
Eutrema salsugineum	114	114	0	0
Raphanus sativus	119	119	0	0

Citrus sinensis	331	180	41	110
Citrus trifoliata	6	0	6	0

Table S4 Characteristics of MITEs superfamily

Superfamily	5' TSD	5' TIR	Example of 5' TIR
Tc1/Mariner	TA	Variable	CTCCCTCCCT
PIF/Harbinger	TTA&TAA	BNNNN	GGGCC, TGGTG
Mutator	8-10 bp	SNNNN	GGATA, CAGTA
hAT	8 bp	YANNS	TAGGG, CAGGG
CACTA	2-3 bp	CACTR	CACTA, CACTG
Micron	(TA)n	SNNNN	CAGTA, CAGTT

Table S5 The substitution rates of nucleic acid used for evaluation of MITEs amplification time

Plant clade	Taxa	Substitution rate (sites/year)
Gymnosperms	Pinaceae	1.46E-09
	Gnetophytes	2.91E-08
Angiosperms	Brassicaceae	1.30E-08
	Malvaceae	9.12E-09
	Euphorbiaceae	9.09E-09
	Fabaceae	9.46E-09
	Cucurbitaceae	1.20E-08
	Rosaceae	8.04E-09
	Solanaceae	1.15E-08
	Poaceae	1.21E-08
	others	1.05E-08
Other clades	-	1.30E-08
